# Excessive Kynurenine Metabolism Impairs Lysosomal acidification and Triggers mtDNA Release via the AHR/CISH/ATP6V1A Axis in Decidual Macrophages Associated with Unexplained Recurrent Pregnancy Loss

**DOI:** 10.7150/ijbs.121947

**Published:** 2026-01-01

**Authors:** Guangmin Song, Hongli Li, Man Zhang, Yun Li, Xinyi Tao, Andi Wang, Jianqi Wang, Boris Novakovic, Richard D. Cannon, Richard Saffery, Hongbo Qi, Hua Zhang, Xiaobo Zhou

**Affiliations:** 1 Department of Obstetrics and Gynecology, Chongqing Key Laboratory of Maternal and Fetal Medicine / Joint International Research Laboratory of Reproduction & Development, Ministry of Education / The Innovation and Talent Recruitment Base of Maternal-Fetal Medicine, The First Affiliated Hospital of Chongqing Medical University, No.1 Youyi Rd, Yuzhong District, Chongqing, 400016, China.; 2 Molecular Immunity, Murdoch Children's Research Institute and Department of Paediatrics, University of Melbourne, Melbourne, VIC, Australia.; 3 Department of Oral Sciences, Sir John Walsh Research Institute, Faculty of Dentistry, University of Otago, Dunedin, New Zealand.

**Keywords:** recurrent pregnancy loss, decidual macrophages, aryl hydrocarbon receptor, extravillous trophoblasts, mitochondrial DNA

## Abstract

Metabolic disturbances of decidual macrophages (dMφs) may contribute to the pathology of miscarriage, yet the underlying mechanisms remain poorly defined. Here, we document upregulated tryptophan metabolic pathway in dMφs from women with unexplained recurrent pregnancy loss (URPL), with increased kynurenine (KYN) levels in the decidua and elevated aryl hydrocarbon receptor (AHR) expression in dMφs. Excessive activation of the KYN-AHR axis compromises both mitochondrial and lysosomal integrity. This impairment facilitates the leakage of mtDNA into the cytoplasm and subsequent release into the extracellular space, thereby activating the cGAS-STING signaling cascade. Mechanistically, AHR directly binds to the xenobiotic response element within the *CISH* promoter region, promoting its transcription. The upregulation of CISH promotes the ubiquitination and degradation of ATP6V1A, disrupting lysosomal acidification and exacerbating mtDNA release. *In vivo*, excessive administration of KYN in pregnant mice increases the rate of embryo resorption, whereas pharmacological inhibition of AHR partially attenuates cGAS-STING pathway activation in dMφs and ameliorates fetal loss in an abortion-prone mouse model. Collectively, our findings describe a pivotal role for the AHR/CISH/ATP6V1A axis in orchestrating immune dysfunction within the decidua that may contribute to URPL, which sheds new light on the potential pathogenesis of URPL and paves the way for improving pregnancy outcomes.

## Introduction

Recurrent pregnancy loss (RPL), defined as two or more consecutive pregnancy losses before 24 weeks of gestation, affects approximately 5% of women of reproductive age 1. While known causes include chromosomal abnormalities, uterine anomalies, infections, and endocrine disorders, nearly half of all cases lack an identifiable etiology, and are classified as unexplained RPL (URPL) 2. Histopathological studies have consistently implicated abnormalities within the decidual microenvironment as potential contributors to URPL 3. The decidua is composed of maternal immune cells, decidual stromal cells, and fetal extravillous trophoblasts (EVTs) 4. It functions not only as the structural interface for maternal-fetal nutrient and gas exchange but also as a critical immunoregulatory hub, maintaining the delicate equilibrium between promoting fetal development and ensuring maternal immune tolerance 5. Among the various proposed causes of URPL, excessive inflammation has emerged as a potentially significant pathogenic factor 6. The decidual immune landscape is highly heterogeneous, encompassing both pro-inflammatory and anti-inflammatory immune cell populations 7. The specific immune cell types that drive pregnancy loss in the context of decidual dysfunction remain incompletely understood.

Maternal immune tolerance toward the semi-allogeneic fetus is intricately regulated by decidual immune cells 8, 9. Among them, decidual macrophages (dMφs), comprising approximately 20-30% of the total immune cells in the decidua, play a central role in sustaining a healthy pregnancy 10. During early gestation, dMφs contribute to placental development by removing apoptotic cells, modulating trophoblast invasion, and promoting spiral artery remodeling 11. In addition, dMφs maintain immunological equilibrium at the maternal-fetal interface by secreting a balanced array of anti-inflammatory (e.g., IL-10, TGF-β) and pro-inflammatory cytokines (e.g., TNF-α, IL-6) 12. The critical importance of these cells is further underscored by evidence showing that dMφs depletion post-conception leads to fetal resorption and implantation failure 13, 14. Recent studies have drawn attention to the involvement of dMφs in the pathogenesis of URPL, particularly through metabolic reprogramming mechanisms. In the decidua of URPL pregnancies, lactate accumulation activates the HIF-1α/SRC/LDHA axis in dMφs, promoting glycolysis and leading to a pro-inflammatory phenotype that compromises maternal-fetal tolerance 15. Furthermore, imbalances in fatty acid metabolism, characterized by enhanced fatty acid uptake and CPT1A deficiency, result in impaired mitochondrial β-oxidation, lipid droplet accumulation, and increased pro-inflammatory cytokine secretion by dMφs 16. These disturbances collectively disrupt immune balance homeostasis, hinder trophoblast invasion, and impair spiral artery remodeling, ultimately culminating in fetal development failure or miscarriage. Although the functional significance of dMφs in URPL is well-established, the precise regulatory mechanisms, and molecular signaling pathways, remain obscure.

In this study, we investigated the impact of abnormal tryptophan metabolism in dMφs in URPL. By integrating single-cell RNA sequencing (scRNA-seq), multiplex cytokine analysis, targeted metabolomics, *in vitro* cell experiments and *in vivo* mouse models, we demonstrated that aberrant activation of the kynurenine (KYN) pathway skews dMφs toward an immunologically activated state through aryl hydrocarbon receptor (AHR)-mediated activation of the cGAS-STING pathway. Through combined epigenomic and transcriptomic analyses, we identified cytokine inducible SH2 containing protein (CISH) as a direct transcriptional target of AHR. Mechanistically, we showed that the KYN/AHR/CISH cascade promotes the ubiquitination and degradation of ATP6V1A, impairs lysosomal acidification and precipitates mitochondrial DNA (mtDNA) leakage. The extracellular release of mtDNA then destabilizes maternal-fetal immune homeostasis by compromising EVTs function via paracrine signaling. Pharmacological inhibition of AHR could ameliorate fetal loss in an abortion-prone (AP) mouse model. Collectively, our findings uncover a previously unrecognized mechanism linking dysregulated tryptophan metabolism in dMφs to URPL pathogenesis and identify novel therapeutic targets for clinical intervention.

## Materials and Methods

### scRNA-seq analysis

Publicly available scRNA-seq datasets, GSE214607 17 and PRJNA672658 18, were obtained from the GEO and SRA repositories. Raw FASTQ files were aligned to the GRCh38 human genome using Cell Ranger (RRID:SCR_017344) to generate gene-cell count matrices. Quality control involved removing doublets by DoubletFinder (RRID:SCR_018771) and filtering out cells with a mitochondrial gene content exceeding 10%, with fewer than 200 or greater than 8,000 genes detected, and filtering out genes expressed in fewer than three cells. A total of 132,301 high-quality cells were retained for downstream analysis. Normalization, scaling, and principal component analysis (PCA) were conducted using the Seurat (RRID:SCR_016341). Batch effects across datasets were corrected with harmony integration (group.by.vars = "orig.ident", dims.use = 1:30), followed by uniform manifold approximation and projection (UMAP) for dimensionality reduction and unsupervised clustering (resolution = 0.5). Clusters were annotated using canonical markers identified through differential gene analysis via the wilcoxon rank-sum test (FindAllMarkers). To evaluate the contribution of cell types to phenotypic differences, effect sizes were ranked using AUGUR 19 package, which calculates area under the curve (AUC) values through cross-validated logistic regression.

dMφs subclusters extracted from the annotated dataset were analyzed for differentially expressed genes (DEGs) between URPL and normal pregnancies using the wilcoxon test (FindMarkers; |log2FC| > 0.25, adjusted *p* < 0.05). Gene Set Enrichment Analysis (GSEA) against Kyoto Encyclopedia of Genes and Genomes (KEGG) pathways identified biologically relevant signatures, with normalized enrichment scores (NES) and false discovery rates (FDR) computed to determine statistical significance.

To enhance the robustness of metabolic profiling, dMφs transcriptomes were aggregated into metacells (SuperCell v1.0; merging ratio = 100:1), reducing sparsity while maintaining cell-type specificity. These metacells were mapped to the genome-scale metabolic model in COMPASS 20, which quantifies pathway activities via linear optimization of reaction flux states. Differential metabolic activity between URPL and normal pregnancies was evaluated using wilcoxon rank-sum tests with Benjamini-Hochberg adjustment (FDR < 0.05), and the results were visualized as network-score scatterplots generated by COMPASS's built-in plotting functions.

### Clinical sample collection

Decidual tissue samples were collected from 30 patients with URPL and 30 patients with normal pregnancies who underwent pregnancy termination for non-medical reasons. This study was performed following the principles of the Declaration of Helsinki and approved by the First Affiliated Hospital Ethics Committee of Chongqing Medical University (No. 2021-746). Exclusion criteria included parental or embryonic chromosomal abnormalities, female genital tract abnormalities, history of infection, endocrine disorders, and other known causes of miscarriage. Baseline characteristics of both groups are provided in Table S1. Following collection, all samples were placed in ice-cold phosphate-buffered saline (PBS) within sterile containers and promptly transported to the laboratory for further processing.

### Targeted metabolomics

Tryptophan-targeted metabolomic analysis was performed on 20 pretreated human decidual tissue samples (10 from URPL pregnancies and 10 from normal pregnancies). The quantification of tryptophan metabolites in URPL and normal decidua was analyzed by Shanghai Biotree Biomedical Technology (Shanghai, China). Approximately 50 mg of each preprocessed decidual tissue sample was homogenized in 500 μL of precooled extraction buffer (methanol:acetonitrile:water = 2:2:1, v/v/v, containing 0.1% formic acid and isotope-labeled internal standards), followed by vortexing, grinding, and ultrasonic treatment. After protein precipitation at -40 °C and centrifugation (12000 rpm, 15 min), the supernatant was collected, evaporated, reconstituted with 80 μL of a 0.1% formic acid aqueous solution, and subjected to UHPLC-MS/MS analysis using a Vanquish Flex UHPLC system with a Waters ACQUITY UPLC HSS T3 column. Mobile phase A consisted of 0.1% formic acid in water, while mobile phase B consisted of 0.1% formic acid in acetonitrile. Detection was performed using a SCIEX Triple Quad™ 6500+ mass spectrometer operating in multiple reaction monitoring (MRM) mode. Data processing was performed using SCIEX Analyst and MultiQuant software. Metabolites with > 50% missing values were excluded, and missing values were imputed with a random fraction (10-50%) of the minimum detected value. OPLS-DA, conducted in SIMCA (RRID:SCR_014688) after log transformation and UV scaling, assessed metabolic differences, with model significance evaluated via permutation tests (n = 200). Differential metabolite levels were analyzed using Student's t-test (*p* < 0.05).

### Immunofluorescence

Paraffin-embedded decidual tissue sections were heated at 65°C for 5 h, deparaffinized with xylene, and rehydrated through a graded ethanol series. Antigen retrieval was performed using sodium citrate buffer and microwave heating, followed by washing with PBS and blocking with 5% BSA for 1 hour. The sections were then incubated overnight at 4°C with diluted primary antibodies, washed with PBS, and incubated with fluorescent secondary antibodies at room temperature for 1 h in the dark. Nuclei were stained with DAPI for 10 min, and after a final PBS wash, the slides were mounted with an anti-fade reagent. Fluorescence images were captured using a fluorescence microscope and analyzed with ImageJ software.

### Isolation of human dMφs

Decidual tissue was washed with cold PBS, minced into pieces and digested at 37 °C for 1 h with 0.1% collagenase IV (Millipore Sigma, Cat#C5138) and 0.01% DNase I (Millipore Sigma, Cat#10104159001). The suspension was filtered through 100, 200, and 400 mesh nylon filters, and mononuclear cells were collected via ficoll density gradient centrifugation (1,000 × g, 20 min; GE Healthcare, Cat#17144002). CD14⁺ dMφs were isolated using anti-human CD14 microbeads (Miltenyi Biotec, Cat#130-050-201) following the manufacturer's instructions. Human dMφs were cultured in RPMI 1640 with 10% FBS and 1% penicillin-streptomycin.

### Flow cytometry analysis

Mononuclear cells were resuspended in 100 μL PBS and stained with 0.1 μL Fixable Viability Stain 450 (BD Biosciences Cat# 562247, RRID: AB_2869405), followed by incubation at room temperature in the dark for 15 min. After washing with 1 mL PBS and centrifugation at 300 g for 5 min, the supernatant was discarded. The cells were resuspended in 100 μL staining buffer and incubated with 1 μL each of FITC anti-human CD14 (BioLegend Cat# 982502, RRID: AB_2616906) and APC-Cy7 anti-human CD45 (BioLegend Cat# 304014, RRID: AB_314402) antibodies at 4°C in the dark for 30 min. After another wash with 1 mL staining buffer and centrifugation, the cells were fixed in 100 μL fixation buffer at room temperature in the dark for 30 min. The samples were then permeabilized by washing twice with 1 mL permeabilization buffer (PB) and resuspending in 100 μL PB containing 1 μL PE-Cy7 anti-human AHR antibody (Thermo Fisher Scientific Cat# 25-5925-82, RRID: AB_2573501), followed by incubation at room temperature in the dark for 30 min. After two additional washes with PB, the cells were resuspended in 500 μL PB and analyzed by flow cytometry. To maintain consistency with prior studies in the field 16, 21, we refer to the CD45⁺CD14⁺ subset as dMφs throughout the manuscript. Given that CD14 is expressed by both monocytes and macrophages, this gate yields a macrophage-enriched population that may include a small monocyte fraction.

### Mouse model

All animal procedures were performed in compliance with the Guide for the Care and Use of Laboratory Animals (China) and were approved by the Animal Care and Use Committee of Chongqing Medical University (No. 2022185). To establish a NP mouse model, female CBA/J mice were mated with male BALB/c mice, while the AP mouse model was generated by mating female CBA/J mice with male DBA/2 mice. The detection of a vaginal plug was considered as gestational day (GD) 0.5. Pregnant AP model mice were assigned to two groups: one receiving daily intraperitoneal injections of corn oil (AP group) and the other treated with CH223191 (20 µg/kg, MedChem Express, Cat#HY-12684) via intraperitoneal injection (AP + CH223191 group) from GD 6.5 to GD12.5. Pregnant C57BL/6 mice were injected intraperitoneally with KYN (10 mg/kg, MedChem Express, Cat#HY-104026) or PBS from GD 0.5 to GD 12.5. All mice were euthanized at GD 13.5, and embryo resorption rates were recorded. Uterine and embryonic tissues were collected, with a portion fixed in 4% paraformaldehyde for immunofluorescence analysis.

### Cell line culture

The THP-1 cell line (RRID: CVCL_0006) was purchased from Procell (Wuhan, China). Cell line identity was confirmed by short tandem repeat (STR) profiling. THP-1 cells tested negative for mycoplasma contamination and were cultured in THP-1-specific medium (Procell, Cat#CM-0233) at 37 °C in a humidified incubator with 5% CO₂. To generate THP-1-Mφs, cells were stimulated with 100 ng/mL phorbol 12-myristate 13-acetate (PMA; Sigma-Aldrich, Cat#P8139) for 24 h. The expression of canonical macrophage markers (e.g., CD86, CD206) was verified by flow cytometry (Figure S1). THP-1-Mφs are widely recognized as a reliable and simplified model for studying macrophage functions 22, 23 and were used in this study to investigate the downstream mechanisms triggered by KYN in URPL.

The HTR-8/SVneo cell line (RRID: CVCL_7162) was obtained from the American Type Culture Collection (ATCC), and its identity was verified by STR profiling. Cells were confirmed to be free of mycoplasma contamination and maintained in RPMI 1640 medium (Gibco, Cat#C11875500BT) supplemented with 10% fetal bovine serum (Pansera, Cat#ST30-2602) and 1% penicillin-streptomycin (Beyotime, Cat#C0222), at 37 °C in a humidified atmosphere containing 5% CO₂. For co-culture experiments, THP-1-Mφs were seeded into the upper chamber of transwell inserts, while HTR-8/SVneo cells were plated in the lower chamber. After 24 h of co-culture, HTR-8/SVneo cells were harvested for downstream analyses, including western blotting, apoptosis assays, wound healing assays, and invasion assays.

### RNA-seq and data analysis

Total RNA was extracted from THP-1-Mφs transfected with either an AHR-overexpressing plasmid or negative control, and RNA integrity was assessed using the Agilent 2100 Bioanalyzer. cDNA libraries were prepared using the NEBNext Ultra Directional RNA Library Prep Kit for Illumina. Library concentration was quantified using a Qubit 2.0 Fluorometer and qRT-PCR. Following library pooling, sequencing was conducted on the Illumina platform. High-quality reads were aligned to the reference genome using HISAT2. Differentially expressed genes (DEGs) were identified with the thresholds of max FPKM ≥ 1, fold change ≥ 1.5, and adjusted *p*-value ≤ 0.05. Gene ontology (GO) term and KEGG pathway enrichment was performed by GSEA.

### Quantitative Real Time Polymerase Chain Reaction (RT-qPCR)

Total RNA was extracted from THP-1-Mφs using TRIzol® reagent (Invitrogen, Cat#15596026) following the manufacturer's protocol. RNA concentration and purity were measured using a NanoDrop™ 2000 spectrophotometer (Thermo Fisher Scientific). Reverse transcription was performed with the Evo M-MLV RT Kit (Accurate Biology, Cat#AG11728) to generate cDNA. RT-qPCR was carried out using the SYBR Green Premix Pro Taq HS qPCR Kit (Accurate Biology, Cat#AG11701) on a CFX96 Real-Time PCR System (Bio-Rad, USA). The primer sequences are listed in Table S2.

### Western blotting

Total proteins were extracted from samples using RIPA lysis buffer (Beyotime, Cat#P0013B,) supplemented with protease and phosphatase inhibitors (Beyotime, Cat#P1045). Protein concentrations were quantified using a bicinchoninic acid (BCA) protein assay kit (Beyotime, Cat#P0012S) according to the manufacturer's protocol. Equal amounts of protein were separated by SDS-PAGE and electro-transferred to PVDF membranes (Roche, Cat#03010040001). After blocking with 5% (w/v) non-fat milk powder in Tris Buffered Saline with 0.1% Tween 20 (TBST) for 1 h at room temperature, membranes were incubated with primary antibodies diluted in blocking buffer overnight at 4°C. Following three washes with TBST, membranes were incubated with appropriate HRP-conjugated secondary antibodies (1:10,000 in blocking buffer) for 1 h at room temperature. Protein bands were visualized using enhanced chemiluminescence substrate and quantified with the Fusion FX5 imaging system (Vilber Lourmat). All western blotting experiments were carried out with three independent biological replicates. Representative immunoblot images are presented, and antibody specifications are provided in Table S3.

### Multiplex cytokine analysis

Cell culture supernatants were collected from human dMφs or THP-1-Mφs following 24 h incubation in 6-well plates (1×10⁶ cells/well). Supernatant cytokine profiling was performed using the AB-plex Human 15-Plex Custom Panel on an ABplex-100 multicolor flow cytometry system, quantifying 15 cytokines: IL-1β, CCL3, GM-CSF, IFN-γ, IL-2, IL-4, IL-5, IL-6, IL-10, IL-17A, CCL4, IL-8, IL-23p40, TNF-α, and IL-2RA. Following bead-based capture and PE-conjugated detection, fluorescence signals were acquired and analyzed via four-parameter logistic regression for concentration determination.

### Transmission electron microscopy

THP-1-Mφs were fixed with 3% glutaraldehyde in 0.2 M phosphate buffer at 4°C for 2 h, followed by post-fixation with 1% osmium tetroxide in the same buffer for an additional 2 h. After graded ethanol dehydration, samples were subjected to propylene oxide infiltration and embedded in Epon 812 resin. Ultrathin sections (70 nm) were prepared using a Leica EM UC7 ultramicrotome with diamond knives, double-stained with uranyl acetate and lead citrate, and imaged using an FEI Tecnai G2 F30 transmission electron microscope (Thermo Fisher Scientific, USA).

### Measurement of mitochondrial function

THP-1-Mφs were processed for mitochondrial ROS detection using 5 μM MitoSOX Red (Thermo Fisher Scientific, Cat#M36007), and for mitochondrial membrane potential assessment with JC-1 (Beyotime Biotechnology, Cat#C2006), with incubations conducted at 37°C for 30 min in the dark to preserve fluorophore integrity. For nuclear visualization, cells were counterstained with Hoechst 33342 (1 μg/mL; Thermo Fisher Scientific, Cat#62249) for 10 min. Fluorescence imaging was subsequently performed using an Olympus IX83 microscope (Olympus, Japan).

### Lysosomal acidification and cathepsin B activity assay

To examine lysosomal acidification, LysoSensor Green DND-189 (Thermo Fisher Scientific, Cat#A66436), a dye that fluoresces in an acidic environment (pH ≤ 5.2), was employed. THP-1-Mφs were loaded with 1 μM LysoSensor in pre-warmed medium at 37 °C for 1 h and then washed twice with PBS. The cells were immediately analyzed by flow cytometry. Alternatively, after counterstaining the cell nuclei with Hoechst 33342, fluorescence imaging was performed using an Olympus IX83 microscope.

To analyze lysosomal hydrolase activity, the Cathepsin B Activity Assay Kit (Abcam, Cat#ab65300) was used according to the manufacturer's instructions. A total of 5×10⁶ THP-1-Mφs were harvested and resuspended in 500 μL of lysis buffer. After incubating on ice for 30 min, the cells were centrifuged, and the supernatant was collected. Protein concentration was determined, and 50 μL of the protein solution (200 μg) was transferred into a black 96-well microtiter plate. Substrate and reaction buffer were added, and the plate was incubated at 37°C for 90 min. Fluorescence was then measured with excitation and emission wavelengths of 400 nm and 505 nm, respectively.

### mtDNA quantification

THP-1-Mφs were seeded in 6-well microtiter plates at a density of 1 × 10⁶ cells per well in 2 mL of complete medium and cultured for 24 h. The culture supernatants were then collected, and total extracellular DNA was extracted using the DNeasy® Blood & Tissue Kit (Qiagen, Cat#69504) according to the manufacturer's instructions. The relative copy number of mtDNA was quantified by RT-qPCR using the Human Mitochondrial DNA Monitoring Primer Set (Takara, Cat#BA0001), following the manufacturer's protocol.

### Cleavage Under Targets and Tagmentation (CUT&Tag) analysis

Chromatin profiling was conducted using the NovoNGS CUT&Tag 4.0 High-Sensitivity Kit (NovoProtein, Cat#N259-YH01-01A) in strict accordance with the manufacturer's protocol. Briefly, 1×10^5^ cells were collected, washed, and fixed with 1% formaldehyde at room temperature for 10 min, followed by quenching with 2.5 M glycine. Cell membranes were permeabilized using CMPB buffer at 62 °C for 10 min, followed by equilibration in dilution buffer at 37°C for 30 min. After magnetic bead conjugation, chromatin was immune-targeted with AHR-specific antibody (Cell Signaling Technology Cat# 83200, RRID: AB_2800011) overnight at 4 °C, then with secondary antibody at room temperature for 1 h, followed by tagmentation with ChiTag transposase at 37 °C for 1 h. Following fragmentation, crosslinks were reversed with Proteinase K at 50 °C for 2 h. Libraries were amplified using i5 and i7 index primers and 2× HiFi AmpliMix for 30 cycles, purified with magnetic beads, and sequenced on an Illumina platform with 150 bp paired-end reads. Sequencing data were aligned to the GRCh38/hg38 reference genome and *Escherichia coli* K-12 DH10B spike-in genome using Bowtie2 (v2.4.5), followed by spike-in calibration-derived depth normalization. Peak calling performed using MACS2 (q < 0.05) and visualization conducted using IGV (v2.11.9).

### Luciferase reporter assay

The *CISH* promoter region (-392 to +107 bp) enriched by the anti-AHR antibody in the CUT&Tag experiment was cloned into the pGL3-Basic luciferase reporter vector to generate the wild-type construct pGL3-CISH-WT. Potential AHR binding motifs (XRE1: +42 bp; XRE2: -323 bp) were predicted using PROMO and JASPAR databases. Site-directed mutagenesis (CACGC→TATGT) was subsequently performed to generate XRE1-mutant (pGL3-CISH-Mut1) and XRE2-mutant (pGL3-CISH-Mut2) plasmids. The promoter vectors, together with pRL-TK, which contains *Renilla* luciferase as the inner control, were transfected into THP-1-Mφs, with AHR overexpression plasmids or negative control plasmids. Luciferase reporter activity was detected using a dual-luciferase reporter assay system (Promega Corporation, Cat#E1960) according to the manufacturer's protocol and reporter gene activity was determined by normalizing the firefly luciferase activity to *Renilla* luciferase activity.

### Chromatin Immunoprecipitation (ChIP)

The Chromatin Immunoprecipitation (ChIP) assay was performed as previously described 24. Briefly, THP-1-Mφs were fixed with 1% formaldehyde for 10 min at room temperature, followed by quenching with 0.125 M glycine for 10 min at 4 °C. Cells were then pelleted by centrifugation (700 g, 4 °C, 5 min) and processed for chromatin preparation. After cell lysis, genomic DNA was fragmented by sonication to yield 100-300 bp fragments. One percent of the sonicated chromatin was reserved as input control, while the remaining samples were subjected to immunoprecipitation overnight at 4°C using either AHR-specific antibody or Rabbit polyclonal IgG isotype control antibody (Proteintech Cat# 10284-1-AP, RRID: AB_2877729). Immunocomplexes were captured using pre-blocked protein A/G magnetic beads (Thermo Fisher Scientific, Cat#88803). Following five sequential washes, bound DNA-protein complexes were eluted, reverse crosslinked, and purified using the QIAquick PCR Purification Kit (QIAGEN, Cat#A-28106). DNA enrichment was quantified by RT-qPCR with TB Green Premix Ex Taq II (Takara, Cat#RR820A) on a 7500 Real-Time PCR System. The CISH promoter-specific primers used were: Forward, 5'-GCCCTGAGCAGTGAAAGGAA-3'; Reverse, 5'-CTTCAGCGTCGCGATTGGTC-3'.

### Co-immunoprecipitation

Co-immunoprecipitation (Co-IP) was performed using protein A/G magnetic beads (Selleck Chemicals, Cat#23201) according to the manufacturer's instructions. Cultured cells were harvested, resuspended in IP lysis buffer (Beyotime, Cat#P0013), and incubated for 1 h with gentle agitation. Cell lysates were clarified by centrifugation (13000 rpm, 4°C, 20 min), with 1% of the supernatant reserved as input control. The remaining supernatant was incubated overnight at 4°C with specific antibodies. Immune complexes were captured by incubating with pre-washed protein A/G magnetic beads for 4 h at 4°C. Following three washes with ice-cold lysis buffer, bound proteins were eluted and analyzed by western blotting. Antibody specifications are listed in Table S3 (Supporting Information).

### Isolation of primary EVTs

Human primary EVTs isolation was performed as previously described 25. Briefly, villous tissue was gently dissected from the basal membrane and digested at 37 °C for 8 min using a trypsin (0.2%) solution containing EDTA (0.02%). Digestion was terminated with DMEM/F12 supplemented with 10% FBS. The resulting cell suspension was sequentially filtered through mesh nylon screens and subjected to density-gradient centrifugation on Ficoll (GE Healthcare, Cat#17144002) at 800 × g for 20 min. Collected cells were washed and incubated with PE-conjugated anti-HLA-G antibody (Abcam, Cat# ab24384) to identify primary EVTs, after which HLA-G⁺ cells were isolated using Anti-PE MicroBeads (Miltenyi Biotec, Cat#130-048-801). Purified primary EVTs were then co-cultured with dMφs in a 0.4-μm-pore transwell system, with dMφs seeded in the upper chamber for 24 h. Following co-culture, primary EVTs in the lower chamber were collected for protein extraction and subsequent functional assays.

### Culture of hTSCs and EVTs differentiation

The procedures for human trophoblast stem cells (hTSCs) culture and EVTs differentiation were performed as previous reported 26. Briefly, hTSCs were cultured in 6-well plates pre-coated with 5 μg/ml collagen type IV. The hTSCs culture medium consisted of DMEM/F12 supplemented with 0.2% FBS (ThermoFisher, Cat#10099141), 0.5% penicillin-streptomycin (PS, ThermoFisher, Cat#15140122), 0.1 mM 2-mercaptoethanol (ThermoFisher, Cat#21985023), 0.3% bovine serum albumin (BSA, Sigma, Cat# A9418), 1% Insulin-Transferrin-Selenium-X (ITS-X, BasalMedia, Cat#S452J7), 0.5 μM A83-01 (MedChemExpress, Cat#HY-10432A), 1 μM SB431542 (MedChemExpress, Cat#HY-10431), 5 μM Y27632 (Selleck, Cat#S1049,), 0.8 mM valproic acid (MedChemExpress, Cat#HY-10585), and 2 μM CHIR99021 (Selleck, Cat#S1263). When the hTSCs reached approximately 80% confluence, they were dissociated and passaged using TrypLE (Thermo Fisher Scientific, Cat#12604021). To induce hTSCs-derived EVTs (hTSCs-EVTs), hTSCs were seeded onto 6-well plates pre-coated with 1 μg/ml collagen IV and cultured in 2 mL of EVTs medium [DMEM/F12 supplemented with 0.1 mM 2-mercaptoethanol, 0.5% PS, 0.3% BSA, 1% ITS-X, 100 ng/ml NRG1 (CST, Cat#26941), 7.5 μM A83-01, 2.5 μM Y27632, and 4% KnockOut Serum Replacement (KSR, ThermoFisher, Cat#10828010)]. After plating, Matrigel (Corning, Cat#356231) was added to a final concentration of 2%. On day 3, the medium was replaced with EVTs medium lacking NRG1, and the Matrigel concentration was reduced to 0.5%. When the cells reached approximately 80% confluence on day 6, they were passaged at a 1:2 split ratio and cultured for an additional two days in EVTs medium without NRG1 and KSR. Then, hTSCs-EVTs were co-cultured with dMφs in a 0.4-μm-pore transwell system (Corning, Cat#3412), with dMφs seeded in the upper chamber for 24 hours. After co-culture, the hTSCs-EVTs were collected for immunofluorescence analysis.

### Apoptosis analysis

HTR-8/SVneo cells were treated according to the designated experimental conditions. Following treatment, cells were harvested by trypsinization, washed, and stained with Annexin V-FITC and Propidium Iodide (PI) using the Annexin V-FITC/PI Apoptosis Detection Kit (Beyotime, Cat#C1383L). Staining was performed in 1× binding buffer at room temperature in the dark for 30 min. Apoptotic cells were then quantified by flow cytometry.

### Cell invasion assay

Transwell inserts (Corning, Cat#3428) were pre-coated with 80 μL of Matrigel (BD Biosciences, Cat#356234) diluted 1:8 in serum-free medium. A total of 5 × 10⁴ cells were resuspended in 200 μL of serum-free medium and seeded into the upper chamber. The lower chamber was filled with 600 μL of medium containing 10% fetal bovine serum as a chemoattractant. After 48 h incubation at 37 °C, non-invading cells and Matrigel on the upper surface of the membrane were gently removed with a cotton swab. Invaded cells on the lower surface were fixed with 4% paraformaldehyde for 15 min and stained with crystal violet for 30 min. Images were captured using a light microscope (EVOS FL Auto Imaging System, Thermo Fisher Scientific, USA), and the invasion rate was quantified using ImageJ software.

### Wound healing assay

HTR-8/SVneo cells were seeded into 6-well microtiter plates and cultured until reaching 90% confluence. A linear wound was introduced in the cell monolayer using a sterile 10 μL pipette tip. Floating cells and debris were removed by washing twice with PBS. Cells were then incubated in serum-free medium at 37 °C, and wound closure was monitored by capturing images at 0 h and 24 h using a light microscope. The wound area was quantified using ImageJ software, and the migration rate was calculated as the percentage of wound closure over time.

### Statistical analysis

Statistical analysis was performed using GraphPad Prism software (version 8.0). Data are presented as mean ± standard deviation (SD). Comparisons between two groups were performed using an unpaired, two-sided Student's t-test, with *p* < 0.05 considered statistically significant. For comparisons involving multiple conditions, one-way analysis of variance (ANOVA) was used.

## Results

### dMφs exhibit enhanced kynurenine pathway activity in URPL pregnancies

**Figure 1:**
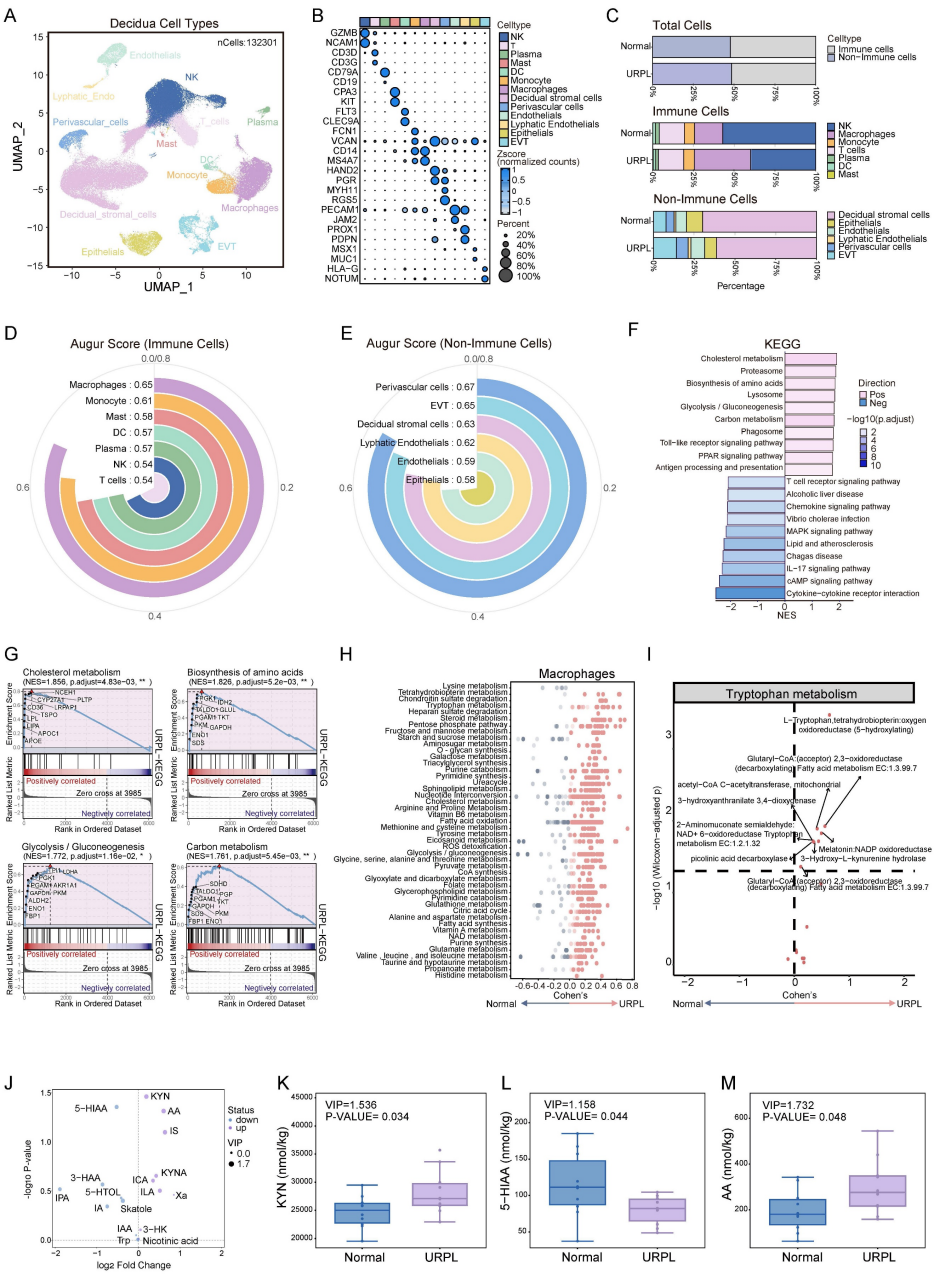
**Multi-omic profiling reveals metabolic dysregulation in decidual macrophages (dMφs) from URPL pregnancies.** (**A**) UMAP visualization of 132,301 decidual tissue cells from 9 URPL and 10 normal pregnancies. (**B**) Bubble chart illustrating the expression levels of canonical marker genes used to annotate specific cell populations. (**C**) Bar plots illustrating: (i) the proportions of immune and non-immune cells among total decidual cells; (ii) the composition of immune cell subsets within immune cells, and (iii) the composition of non-immune cell subsets within non-immune cells in URPL and normal pregnancies. (**D-E**) AUGUR scores quantifying cell type-specific transcriptional perturbations in immune (**D**) and non-immune (**E**) populations, highlighting the cell types most responsive to the pathological microenvironment of decidua from URPL pregnancies. (**F**) Bar plot of the top 10 most significantly upregulated and downregulated KEGG pathways in dMφs from URPL pregnancies. (**G**) GSEA analysis of pathways related to cholesterol metabolism, amino acid biosynthesis, glycolysis/gluconeogenesis, and carbon metabolism. (**H**) COMPASS analysis revealing differential metabolic pathway activities in dMφs between URPL and normal pregnancies. The X-axis represents Cohen's d values; dot color intensity reflects statistical significance. (**I**) Altered activity of the tryptophan metabolism pathway in dMφs from URPL pregnancies. (**J**) Volcano plot showing differentially abundant tryptophan metabolites in decidual tissues, between URPL and normal pregnancies, identified through targeted metabolomics. Purple dots represent upregulated metabolites, blue dots represent downregulated metabolites, and the size of each dot corresponds to the VIP value. Abbreviations: KYN: Kynurenine; 5-HIAA: 5-Hydroxyindoleacetic Acid; AA: Anthranilic Acid; IS: Indole Sulfate; KYNA: Kynurenic Acid; ICA: Indole-3-carboxaldehyde; 3-HAA: 3-Hydroxyanthranilic Acid; IPA: Indole-3-propionic acid; IAA: Indole-3-acetic acid; ILA: Indole-3-acetalamine; Xa: Xanthurenic Acid; 5-HTOL: 5-Hydroxytryptophol; Skatole: 3-Methylindole; IA: Indole Acetic Acid; 3-HK: 3-Hydroxykynurenine; Nicotinic Acid; Trp: Tryptophan; 5-M-IAA: 5-Methoxy-3-indoleacetic Acid. (**K-M**) Boxplots comparing the concentrations of KYN (**K**), 5-HIAA (**L**), and AA (**M**) in decidua between URPL and normal pregnancies. Each group includes n = 10 samples; statistical significance was assessed using the Student's t-test.

Abnormal immune cell function within decidua is an important cause of pregnancy loss 8. To delineate the immune alterations associated with URPL, we integrated two single-cell RNA sequencing datasets of first-trimester decidual tissues from normal and URPL pregnancies (Figure S2A-C). This analysis profiled 132,301 decidual cells, clustered into 13 distinct populations comprising 7 immune and 6 non-immune subsets (Figure 1A, B). While the overall abundance of immune cells remained largely consistent between URPL and normal pregnancies, URPL decidua exhibited a substantial expansion of dMφs alongside a reduction in dNK cells (Figure 1C and Figure S2D). Consistent with previous studies 18, 27, 28, analysis of the non-immune compartment revealed an increased proportion of EVTs and a concomitant decline in decidual stromal cells, reflecting the remodeling of decidual tissue in URPL pregnancies (Figure 1C and Figure S2D). To identify cell populations most responsive to the pathological microenvironment of URPL, we employed AUGUR to quantify transcriptional perturbations across decidual cell types. This analysis revealed that dMφs exhibited the highest transcriptional responsiveness among immune populations (Figure 1D), underscoring their pivotal role in URPL pathogenesis. Among non-immune subsets, EVTs and perivascular cells displayed the most significant shifts (Figure 1E).

Since dMφs exhibited the highest transcriptional responsiveness among immune populations associated with URPL, we focused on transcriptomic alterations of dMφs. KEGG pathway enrichment analysis revealed extensive reprogramming of genes involved in metabolic pathways in URPL-associated dMφs, with upregulation of pathways related to cholesterol metabolism, amino acid biosynthesis, glycolysis/gluconeogenesis, and carbon metabolism (Figure 1F, G). COMPASS analysis highlighted dMφs-centric dysregulation in three metabolite hubs: amino acid metabolism (e.g., tryptophan, tyrosine), lipid metabolism (e.g., fatty acid oxidation, triacylglycerol synthesis), and carbohydrate metabolism (e.g., pyruvate metabolism, pentose phosphate pathway) (Figure 1H). Among these, the tryptophan metabolic axis emerged as the most prominently altered amino acid pathway with increased expression of metabolic enzymes associated with L-tryptophan (Figure 1I) in dMφs, suggesting a transcriptionally primed state for altered tryptophan utilization. To determine whether these transcriptomic alterations were reflected at the metabolic level, we performed targeted metabolomic profiling of decidual tissues from normal and URPL pregnancies. Seventeen tryptophan-derived metabolites were quantified in early pregnancy decidua (Figure 1J and Figure S3A-D). URPL pregnancies exhibited a marked elevation of KYN and anthranilic acid, alongside reduced levels of 5-hydroxyindoleacetic acid (5-HIAA), a major serotonin metabolite (Figure 1K-M). These findings indicate a metabolic shift in decidual tryptophan catabolism in URPL pregnancies, characterized by enhanced KYN pathway activation and suppressed serotonin biosynthesis.

### Excessive KYN accumulation upregulates AHR in dMφs associated with URPL

**Figure 2:**
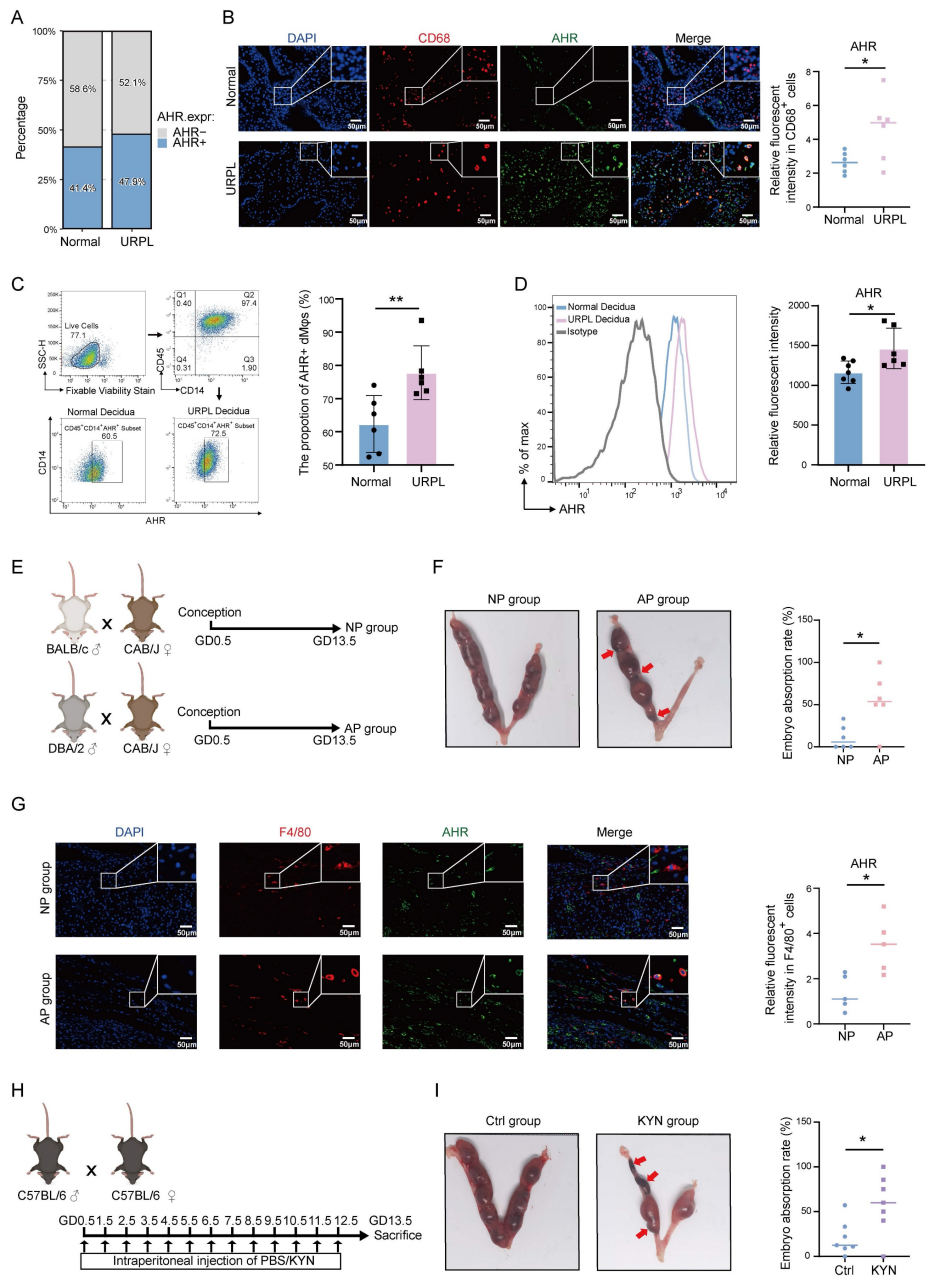
** Upregulated AHR expression in dMφs from URPL pregnancies and abortion prone (AP) Mice.** (**A**) Proportion of AHR-positive dMφs identified from single-cell transcriptomic data of decidual tissues from URPL patients and normal pregnancies. (**B**) Representative immunofluorescence images showing co-staining of AHR and CD68 in decidual tissues from URPL and normal pregnancies (n = 6 per group). (**C-D**) Flow cytometry analysis of dMφs (CD45⁺CD14⁺ subset), quantifying the percentage of AHR-positive cells (**C**) and the relative expression levels of AHR (**D**) in normal and URPL pregnancies (n = 6 per group). (**E**) Schematic overview of the animal study design. CBA/J female mice were mated with BALB/c males to establish a normal pregnancy (NP) model, or with DBA/2 males to generate an AP model. Mice were sacrificed on gestational day (GD) 13.5 to assess embryo resorption rates. (**F**) Representative images showing uterine horns with resorbed embryos indicated by red arrows. Total embryo numbers were recorded (n = 5 per group). (**G**) Immunofluorescence images of AHR and F4/80 co-staining in the decidua from NP and AP mice (n=5 per group), highlighting AHR expression in macrophages. (**H**) Experiment schematic illustrating daily intraperitoneal injections of kynurenine (KYN, 10 mg/kg) or PBS in pregnant C57BL/6J mice from GD 0.5 to GD 12.5. Mice were euthanized on GD 13.5 for embryo assessment. (**I**) Images of uterine horns from KYN- and PBS-treated mice, with resorbed embryos indicated by red arrows. Embryo numbers were recorded (n = 6 per group). Data are presented as mean ± SD. Statistical significance was determined using the Student's t-test; *p < 0.05, **p < 0.01.

Given the observed elevation of KYN in decidua from URPL pregnancies, we next explored whether its downstream effector, AHR, is dysregulated in URPL pregnancies. Single-cell transcriptomics revealed that *AHR* expression was primarily restricted to myeloid immune cell subsets and endothelial cells, with dMφs exhibiting high expression (Figure S4A). Notably, the proportion of AHR⁺ dMφs was higher in URPL pregnancies than in normal pregnancies (Figure 2A). Immunofluorescence staining further confirmed significantly elevated AHR expression level in URPL dMφs (Figure 2B). Consistent with these findings, magnetic-activated cell sorting (MACS) followed by flow cytometry showed a higher frequency and expression level of CD45⁺CD14⁺AHR⁺ dMφs in URPL pregnancies (Figure 2C, D), confirming AHR upregulation in this context. To validate these findings, a spontaneous abortion model (AP) was used. We employed normal pregnancy (NP) and AP mice (Figure 2E), in which the AP mice exhibited a markedly increased embryo resorption rate (Figure 2F). Mirroring the human data, the AHR signal intensity was significantly increased in F4/80⁺ dMφs from AP mice (Figure 2G). To test the functional relevance of AHR activation, pregnant C57BL/6 mice were administered excessive KYN (Figure 2H). Immunofluorescence analysis further showed that KYN-treated mice exhibited significantly increased AHR expression in F4/80⁺ dMφs compared with controls (Figure S4B), confirming effective AHR activation* in vivo*. Excessive KYN treatment significantly increased embryo resorption (Figure 2I), supporting a potential pathogenic role of KYN-induced AHR hyperactivation in pregnancy loss. Together, these findings demonstrate that AHR expression is elevated in dMφs in both URPL pregnancies and AP mice, implicating aberrant AHR signaling in the immunopathogenesis of pregnancy failure.

### AHR promotes inflammatory cytokines secretion and activates the cGAS-STING pathway

**Figure 3:**
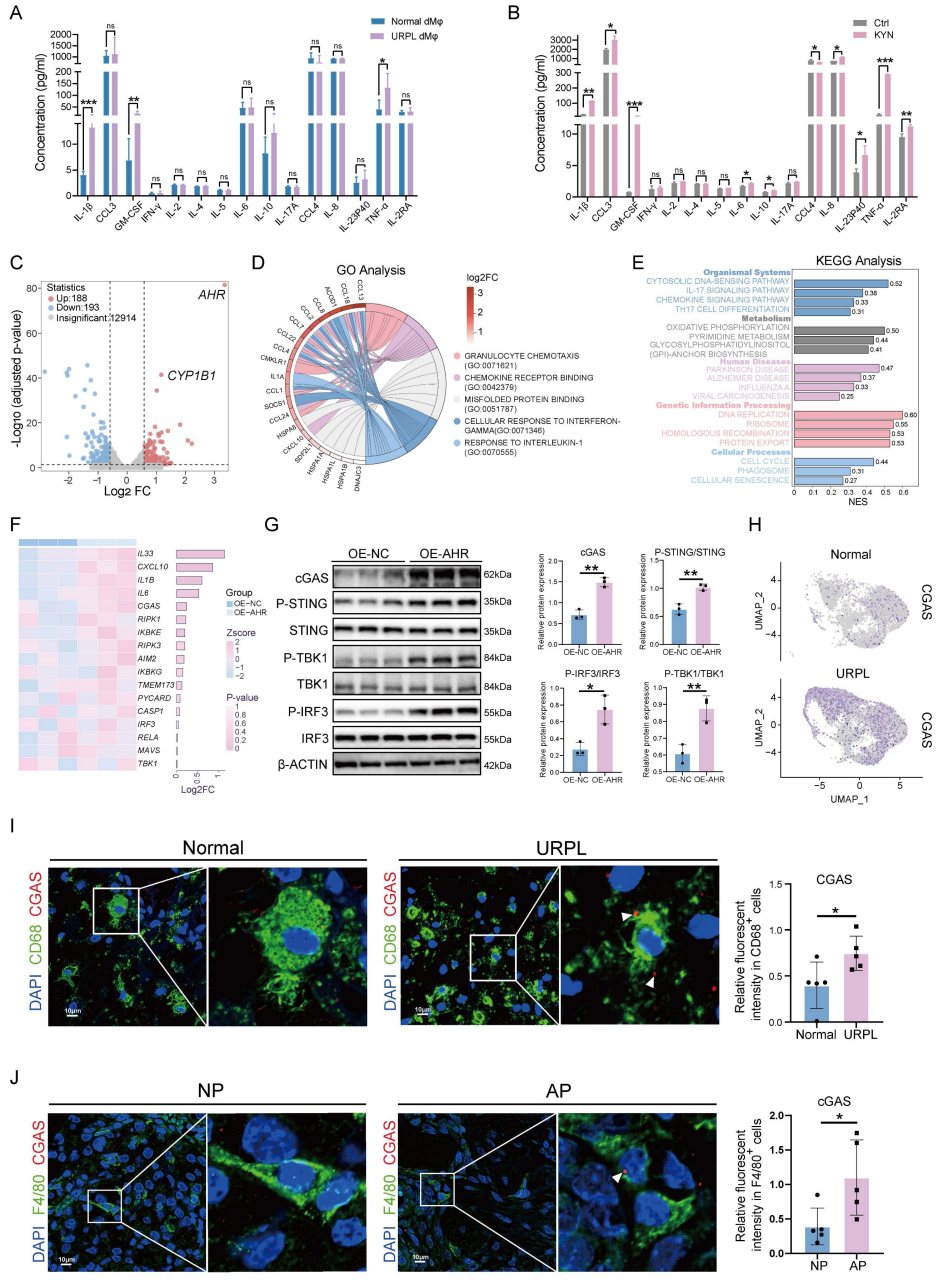
**The KYN-AHR Axis regulates cytokine secretion and activates the cGAS-STING pathway in macrophages.** (**A**) Multiplex analysis of cytokine secretion profiles in dMφs from URPL and normal pregnancies (n = 6 per group). (**B**) Cytokine secretion profiles of THP-1 derived macrophages cells (THP-1-Mφs) treated with or without KYN (25 μM), assessed using a multiplex assay (n = 3 per group). (**C**) RNA-seq analysis comparing gene expression profiles between THP-1-Mφs overexpressing AHR (OE-AHR) and negative control cells (OE-NC). The volcano plot displays significantly differentially expressed genes (n = 3 per group). (**D**) Chord plot showing the top five most significantly enriched GO terms and their associated differentially expressed genes. (**E**) Bar plot presenting the top 20 most significantly enriched pathways from KEGG analysis using GSEA analysis. (**F**) Heatmap and adjacent bar chart depicting the differential expression of genes involved in the “cytosolic DNA-sensing pathway” between OE-AHR and OE-NC cells. (**G**) Western blot analysis of proteins related to the cGAS-STING pathway, including cGAS, p-STING, STING, p-TBK1, TBK1, p-IRF3, and IRF3 in OE-AHR and OE-NC THP-1 cells (n = 3 per group). (**H**) UMAP plot showing cGAS expression levels in dMφs subsets using scRNA-seq data from URPL and normal pregnancies. (**I**) Representative immunofluorescence images showing co-localization of cGAS and CD68 in decidual tissue from URPL and normal pregnancies (n = 5 per group). (**J**) Representative immunofluorescence images of cGAS and F4/80 co-staining in the decidual tissues from NP and AP mice (n = 6 per group). Data are presented as mean ± SD. Statistical significance was determined using the Student's t-test; ns: not significant, *p < 0.05, **p < 0.01, *** *p* < 0.001.

To assess the functional consequences of AHR activation in dMφs, we first examined inflammatory cytokines secretion profiles. Multiplex cytokine assays detected significantly higher levels of IL-1β, GM-CSF, and TNF-α in dMφs from URPL pregnancies than from normal pregnancies (Figure 3A), consistent with the well-documented pro-inflammatory phenotype of dMφs in URPL 21. The influence of the KYN-AHR axis on macrophage cytokine production was further examined by KYN stimulation. Our results revealed that the KYN-induced AHR activation in THP-1-Mφs was associated with robust upregulation of IL-1β, GM-CSF, and TNF-α, alongside modest elevations in the anti-inflammatory cytokine IL-10 and chemokines CCL3 and IL-8 (Figure 3B, Figure S5A). These findings indicate that aberrant activation of the KYN-AHR axis may elicit a mixed immunomodulatory response skewed toward proinflammatory dominance.

To explore the mechanistic link between AHR signaling and macrophage activation, we established THP-1-Mφs with AHR overexpression (OE-AHR), as confirmed by western blotting and RT-qPCR (Figure S5B, C). Transcriptomic profiling identified 381 differentially expressed genes in OE-AHR cells (Figure 3C). GO enrichment analysis pointed to immune-related processes, including responses to interleukin-1 and chemokine signaling (Figure 3D). It was intriguing to discover that the KEGG analysis indicated the activation of the cytosolic DNA-sensing pathway (Figure 3E and Figure S5D). Upregulated transcripts included *CGAS* and downstream effectors such as *IL-6*, *IL-33*, and *CXCL10* were identified in our study (Figure 3F). These gene expression changes were confirmed by RT-qPCR (Figure S5E). In addition, western blotting also confirmed elevated cGAS protein levels, along with enhanced phosphorylation of STING, TBK1, and IRF3 in OE-AHR macrophages (Figure 3G), demonstrating activation of the cGAS-STING signaling pathway upon AHR activation. To assess the clinical relevance of these findings, we examined cGAS expression in decidual tissues. As shown in Figure 3H and Figure 3I, cGAS expression level increased in dMφs from URPL pregnancies. Consistent results were observed in AP mice, where F4/80⁺ dMφs exhibited elevated cGAS expression compared to NP pregnancies (Figure 3J). Together, these findings demonstrate that aberrant AHR activation drives an immunologically activated state through cGAS-STING pathway activation, contributing to immune dysregulation in URPL pregnancies.

### Excessive activation of AHR promotes mtDNA leakage via impairing mitochondrial and lysosomal homeostasis

**Figure 4:**
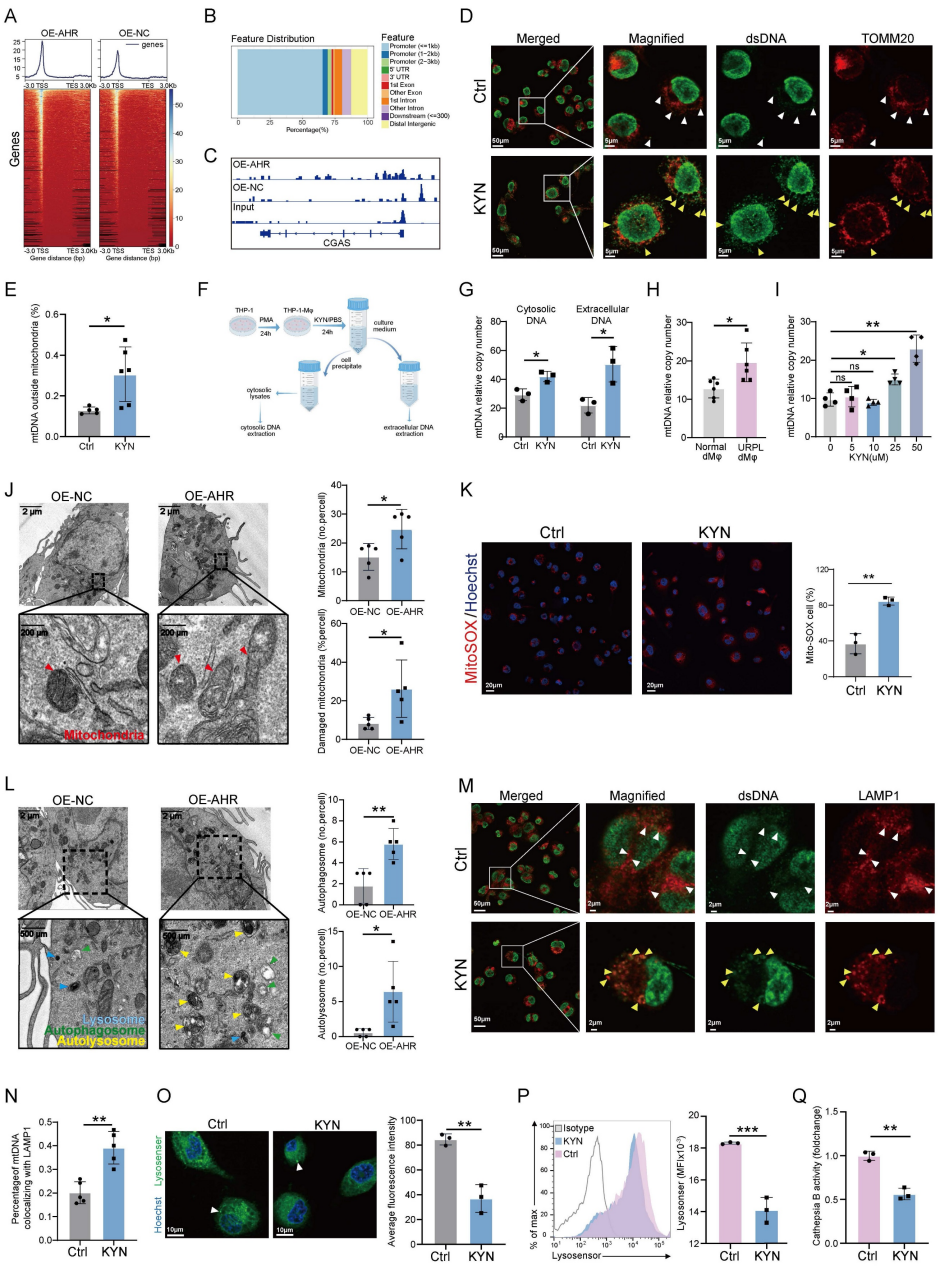
** KYN induces mitochondrial and lysosomal dysfunction, leading to cytosolic and extracellular leakage of mtDNA in macrophages.** (**A**) Heatmap showing the distribution of AHR CUT&Tag binding peaks relative to gene upstream and downstream regions. (**B**) Proportional distribution of AHR binding sites across the functional features of genes. (**C**) Visualization of AHR chromatin binding at the *CGAS* promoter in THP-1-Mφs, displayed using the Integrative Genomics Viewer (IGV). (**D-E**) Representative confocal images (**D**) and colocalization analysis (**E**) of mitochondria (anti-TOMM20) and cytosolic mtDNA (anti-dsDNA) in KYN-treated THP-1-Mφs. White arrowheads indicate mtDNA retained within mitochondria; yellow arrowheads indicate mtDNA released into the cytosol. Images are representative of three independent experiments. (**F**) Experimental workflow for cytosolic and extracellular DNA extraction. (**G**) Relative quantification of mtDNA copy number in cytosolic and extracellular fractions from THP-1-Mφs treated with or without KYN, measured by RT-qPCR (n = 3). ΔCt = Ct(mtDNA) - Ct(nDNA); relative mtDNA copy number = 2^-ΔCt. (**H**) Relative mtDNA copy number in the culture supernatant of primary dMφs from normal and URPL pregnancies, measured by RT-qPCR (n = 6). (**I**) Dose-dependent effects of KYN on mtDNA release from primary dMφs, measured by RT-qPCR (n = 4). (**J**) Representative transmission electron microscopy (TEM) images showing mitochondrial ultrastructure in OE-NC and OE-AHR THP-1-Mφs (n = 5). (**K**) Detection of mitochondrial superoxide production in THP-1-Mφs with or without KYN treatment using MitoSOX staining. (**L**) Representative TEM images showing autophagy-related structures in OE-NC and OE-AHR THP-1-Mφs. (**M-N**) Representative confocal images (**M**) and colocalization analysis (**N**) of lysosomes (anti-LAMP1) and cytosol-released mtDNA (anti-dsDNA) in KYN-treated THP-1-Mφs. White arrowheads indicate mtDNA outside lysosomes; yellow arrowheads indicate mtDNA enclosed within lysosomes. Images are representative of three independent experiments. (**O-P**) Assessment of lysosomal acidity using Lysosensor Green in THP-1-Mφs treated with or without KYN, analyzed by confocal microscopy (**O**) and flow cytometry (**P**). Data represent three independent experiments. (**Q**) Measurement of cytosolic cathepsin B activity in control and KYN-treated THP-1-Mφs. Data are presented as mean ± SD. Statistical significance was determined by the Student's t-test (for comparisons between two groups) or one-way ANOVA (for multiple group comparisons); ns: not significant, *p < 0.05, **p < 0.01, ***p < 0.001.

AHR is a ligand-activated transcription factor involved in immune responses 29. To test the possibility that AHR might regulate *CGAS* transcription, we performed genome-wide mapping of AHR binding sites using CUT&Tag, identifying 2442 peaks. AHR binding was found to be predominantly enriched at promoter regions, with greater signal intensity near transcription start sites in OE-AHR cells than in control cells (Figure 4A, B). However, no significant AHR binding was observed at the *CGAS* promoter (Figure 4C), suggesting that *CGAS* is not a direct transcriptional target of AHR through canonical genomic signaling.

Considering our KEGG analysis indicated the activation of the cytosolic DNA-sensing pathway (Figure 3E and Figure S5D) and the well-established role of mitochondrial DNA (mtDNA) in activating the cGAS-STING pathway 30, we speculated that AHR activation might promote mtDNA leakage. Elevated levels of cytosolic double-stranded DNA (dsDNA) were detected in KYN-treated THP-1-Mφs and were accompanied by a loss of colocalization with mitochondrial markers (Figure 4D, E), indicating a possible cytosolic redistribution of mitochondrial components. To delineate the source of cytosolic dsDNA, DNA was extracted from the cytoplasmic and extracellular fractions of control and KYN-treated THP-1-Mφs (Figure 4F). RT-qPCR of these fractions showed a significant increase in relative mtDNA content (mtDNA/nDNA ratio) in KYN-treated cells (Figure 4G), supporting a mitochondrial origin of cytosolic dsDNA. In primary human dMφs, extracellular mtDNA levels were higher in URPL pregnancies than in normal pregnancies (Figure 4H).

Furthermore, KYN treatment led to a dose-dependent increase in mtDNA release (Figure 4I), which was significantly reduced by AHR inhibition using CH223191 (Figure S6A). These findings suggest that KYN-induced mtDNA release is AHR-dependent. To further validate whether AHR activation induces cGAS-STING signaling in an mtDNA-dependent manner, we treated THP-1 cells with 2',3'-dideoxycytidine (ddC) to induce mtDNA depletion. As shown in Figure S6B, ddC treatment reduced mtDNA levels to less than 10% of baseline. Moreover, ddC treatment attenuated AHR-overexpression-mediated activation of the cGAS-STING pathway (Figure S6C), supporting a mechanistic role for AHR in promoting mtDNA leakage thus inducing cGAS-STING pathway activation. TEM analysis of mitochondrial ultrastructure revealed extensive damage in OE-AHR macrophages, including matrix swelling, disorganization of cristae, and rupture of the outer mitochondrial membrane (Figure 4J). Mitochondrial dysfunction was further supported by increased superoxide production (Figure 4K) and a loss of mitochondrial membrane potential (ΔΨm) (Figure S7A).

In addition, an accumulation of autophagosomes and autolysosomes was observed in OE-AHR THP-1-Mφs (Figure 4L). We next investigated whether this accumulation was linked to mtDNA leakage. Colocalization of dsDNA with lysosomes was significantly increased in KYN-treated cells, while minimal colocalization was observed in untreated cells (Figure 4M, N). Given the critical role of the autophagy-lysosomal pathway in mtDNA clearance, we assessed lysosomal function. Lysosomal acidification was significantly reduced in KYN-treated cells (Figure 4O, P), and lysosomal hydrolase activity was impaired (Figure 4Q). The LC3B-II/I ratio was elevated in OE-AHR cells, indicating autophagosome accumulation (Figure S7B). Inhibition of lysosomal function with bafilomycin A1 abolished differences in LC3B-II levels between the OE-AHR and negative control groups (Figure S7B), confirming that the accumulation of LC3B-II observed was due to impaired autophagic flux rather than increased autophagosome formation. These findings indicate that pathological activation of AHR disrupts mitochondrial and lysosomal homeostasis, leading to mtDNA leakage and subsequent activation of the cGAS-STING pathway.

### AHR induced CISH expression promotes proteasomal degradation of ATP6V1A, disrupts lysosomal acidification

**Figure 5:**
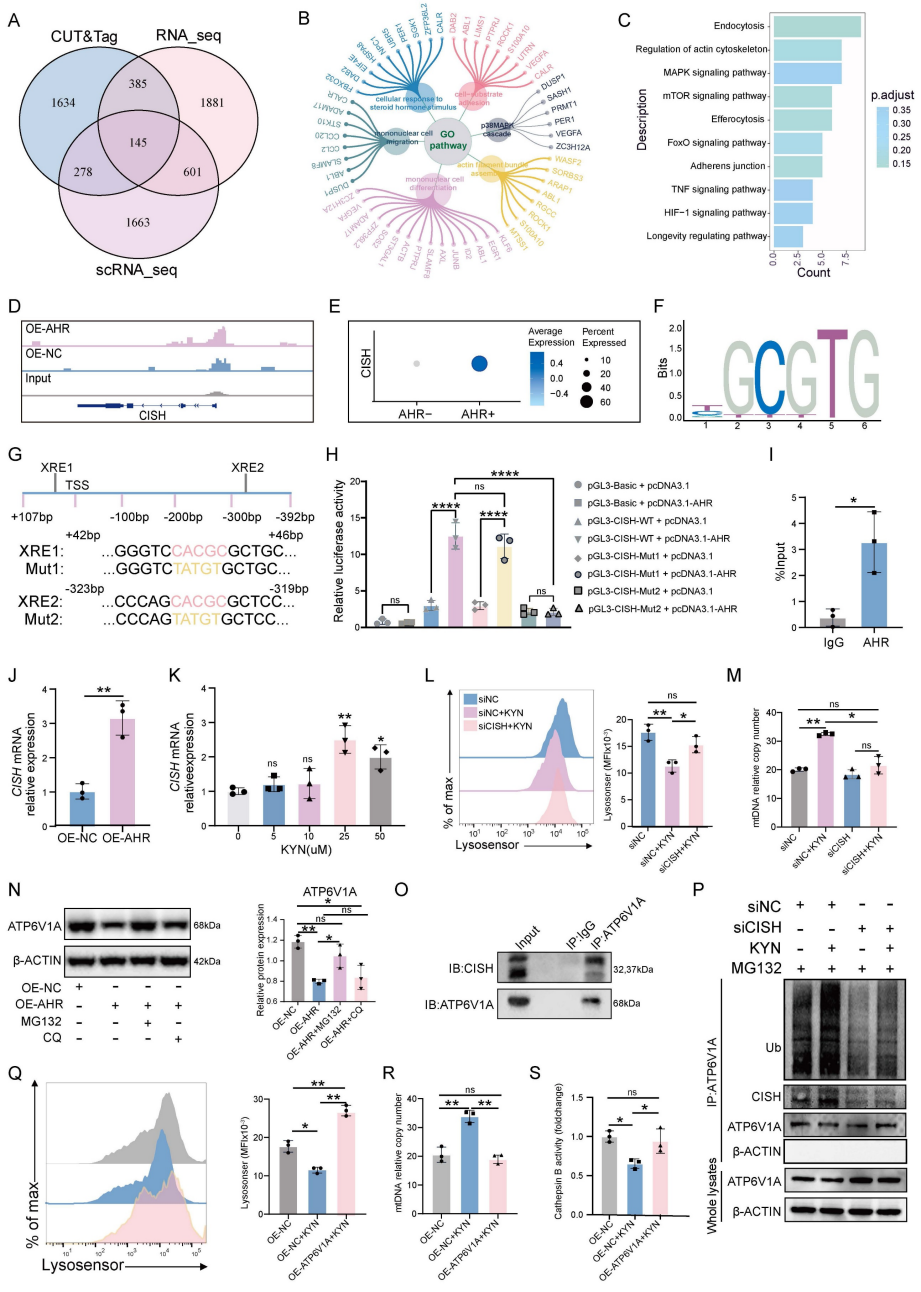
**The AHR-CISH-ATP6V1A axis mediates KYN-induced lysosomal dysfunction in macrophages.** (**A**) Venn diagram showing the overlap of differentially expressed or enriched genes identified from CUT&Tag, RNA-seq, and scRNA-seq datasets. CUT&Tag and RNA-seq data were derived from OE-AHR vs. OE-NC THP-1-Mφs, while scRNA-seq data were obtained using dMφs from URPL pregnancies and normal pregnancies. Differentially expressed genes were selected based on adjusted *p* < 0.05. (**B-C**) GO (**B**) and KEGG (**C**) enrichment analyses of genes common to all three datasets. (**D**) IGV visualization showing AHR chromatin binding at the *CISH* promoter region in THP-1-Mφs. (**E**) Dot plot depicting *CISH* expression in AHR⁻ and AHR⁺ dMφs based on scRNA-seq data. (**F**) Predicted AHR-binding motif retrieved from the JASPAR database. (**G**) Schematic illustration of two predicted AHR-binding elements (XRE1 and XRE2) in the *CISH* promoter, along with their corresponding mutant constructs. (**H**) Dual-luciferase reporter assay assessing the transcriptional activity of *CISH* promoter constructs under various conditions. (**I**) ChIP-qPCR validation of AHR binding at* CISH* promotor region. (**J-K**) RT-qPCR analysis of *CISH* mRNA expression in OE-AHR and OE-NC THP-1-Mφs (**J**), and in dMφs treated with increasing concentrations of KYN (**K**). (**L**) Flow cytometry analysis of lysosomal acidification, measured by Lysosensor Green fluorescence intensity, in THP-1-Mφs transfected with siNC or siCISH and treated with or without KYN. (**M**) Quantification of extracellular mtDNA copy number in culture supernatants of THP-1-Mφs under different treatments, measured by RT-qPCR. (**N**) Western blot analysis of ATP6V1A protein levels in OE-NC and OE-AHR THP-1-Mφs after 6-h treatment with either MG132 (20 μM) or chloroquine (CQ, 50 μM). (**O**) Co-immunoprecipitation assay confirming the interaction between endogenous CISH and ATP6V1A in THP-1-Mφs. CISH was detected as a doublet with molecular weight ~32 and ~37 kDa in the immunoprecipitated samples. (**P**) Ubiquitination assay showing ATP6V1A ubiquitination levels in THP-1-Mφs transfected with siNC or siCISH, and treated with KYN or PBS for 24 h. (**Q**) Flow cytometry analysis of lysosomal pH in OE-NC, OE-NC + KYN, and OE-ATP6V1A + KYN THP-1-Mφs measured with Lysosensor Green. (**R-S**) Quantification of extracellular mtDNA copy number (**R**) and cathepsin B activity (**S**) in indicated groups. All results represent three independent experiments. Data are presented as mean ± SD. Statistical significance was determined using the Student's t-test for two-group comparisons and one-way ANOVA for multiple-group comparisons; ns: not significant, *p < 0.01, **p < 0.01, ***p < 0.001, ****p < 0.0001.

The transcriptional network regulated by AHR in macrophages was profiled through integrative CUT&Tag, RNA-seq, and scRNA-seq analyses, which identified a consensus of 145 differentially expressed genes (Figure 5A). These genes were associated with monocyte migration, steroid hormone responsiveness, and cytoskeletal organization (Figure 5B), suggesting a role for AHR in regulating macrophage motility and the immune microenvironment. Enriched KEGG signaling pathways included MAPK, mTOR, and TNF cascades (Figure 5C), which are all involved in immune responses and cellular stress adaptation 31-33. Among the 145 shared targets, CISH was studied further based on its known involvement in lysosomal function. CISH is a member of the suppressor of cytokine signaling (SOCS) family that functions as a regulator of cytokine signaling and modulates immune cell activation, proliferation, and lysosomal homeostasis 34, 35. AHR binding was observed at the *CISH* promoter region (Figure 5D), and *CISH* mRNA levels were significantly higher in AHR⁺ dMφs than in AHR⁻ counterparts (Figure 5E). Next, we predicted the potential site of AHR binding in the *CISH* promoter region using JASPAR and PROMO databases (Figure 5F and Figure S8A). Two xenobiotic response elements (XREs) within the *CISH* promoter were found (Figure 5G) and designated as XRE1 (+42 bp) and XRE2 (-318 bp). We found that AHR activation enhanced *CISH* promoter activity, and mutation of XRE2 (Mut2), but not XRE1 (Mut1), substantially reduced AHR-induced promoter activation (Figure 5H), indicating that XRE2 is the primary functional site for AHR-driven transcription of *CISH*. ChIP-qPCR further confirmed AHR occupancy at this site (Figure 5I). In OE-AHR THP-1-Mφs, CISH expression was upregulated at both transcript and protein levels (Figure 5J and Figure S8B). KYN treatment of dMφs consistently induced *CISH* expression in a dose-dependent manner (Figure 5K). CISH knockdown in THP-1-Mφs alleviated KYN-induced lysosomal acidification impairment (Figure 5L), reduced accumulation of damaged mitochondrial and autolysosome (Figure S8C), and ameliorated extracellular mtDNA release (Figure 5M). Together, these findings identify *CISH* as a key downstream effector of AHR signaling that mediates mitochondrial-lysosomal dysfunction in macrophages, which may potentially contribute to the mtDNA release.

To elucidate how the AHR-CISH axis impairs lysosomal acidification, we focused on the V-ATPase complex, which drives lysosomal acidification and serves as a known target of CISH 36. CISH has been shown to function as an E3 ubiquitin ligase that promotes the proteasomal degradation of ATP6V1A, the catalytic subunit of V-ATPase, in immune cells, consequently impairing lysosomal acidification 34, 37. Building on our previous finding that AHR-induced lysosomal defects depend on CISH (Figure 5L, M), we hypothesized that the AHR-CISH axis disrupts lysosomal acidification through ATP6V1A regulation in THP-1-Mφs. Consistent with this hypothesis, overexpression of AHR led to a marked reduction in ATP6V1A protein levels without affecting *ATP6V1A* mRNA expression in THP-1-Mφs (Figure S9A, B), suggesting a post-translational regulatory mechanism. To investigate the ATP6V1A degradation pathway, THP-1-Mφs were treated with the proteasome inhibitor MG132 and the lysosomal inhibitor chloroquine. MG132 treatment restored ATP6V1A protein levels, while chloroquine treatment had no significant effect (Figure 5N), indicating that proteasome-dependent degradation of ATP6V1A occurs following AHR activation. Furthermore, CISH knockdown significantly extended the half-life of the ATP6V1A protein (Figure S9C), and co-immunoprecipitation confirmed a direct interaction between endogenous CISH and ATP6V1A (Figure 5O). In addition, KYN stimulation induced polyubiquitination of ATP6V1A, and this effect was completely abolished by CISH silencing (Figure 5P). To assess the functional consequences of ATP6V1A destabilization, we generated THP-1-Mφs overexpressing ATP6V1A. Lysosensor Green staining revealed complete restoration of KYN-induced lysosomal acidification defects (Figure 5Q). Moreover, ATP6V1A overexpression significantly suppressed the release of extracellular mitochondrial DNA and restored lysosomal hydrolase activity, which had been impaired by AHR activation (Figure 5R, S). Together, these findings suggest that CISH acts as the catalytic effector driving the proteasomal degradation of ATP6V1A and ATP6V1A deficiency acts as a critical mediator linking AHR signaling to lysosomal and mitochondrial dysfunction.

### AHR⁺ dMφs derived mtDNA impairs trophoblast function

**Figure 6:**
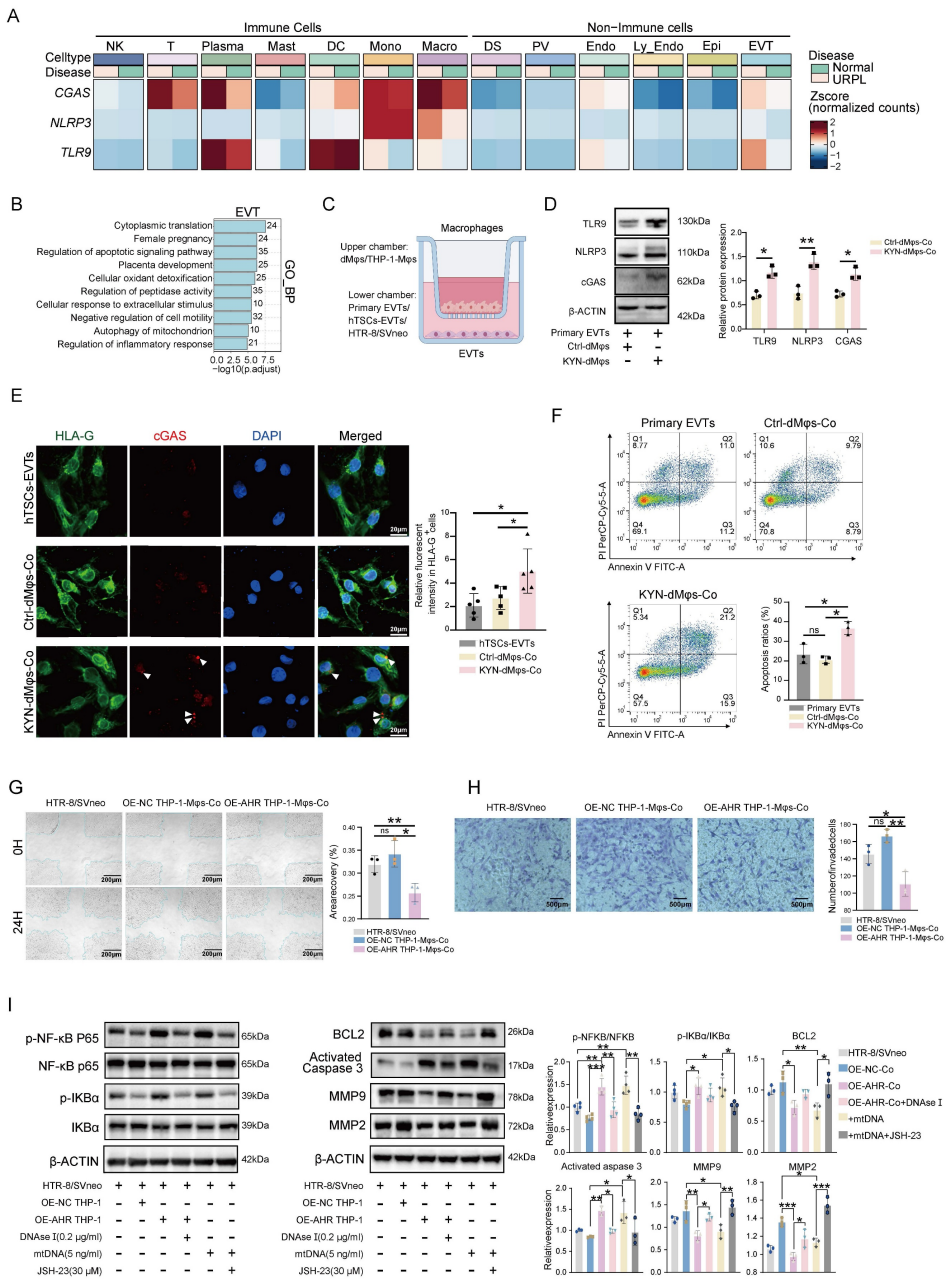
**AHR-activated macrophages impair trophoblast function.** (**A**) Heatmap showing expression levels of *CGAS*, *NLRP3*, and *TLR9* in various cell types determined by scRNA-seq. (**B**) Bar plots displaying significantly enriched GO terms in EVTs from URPL pregnancies. (**C**) Schematic diagram illustrating the co-culture system. (**D**) Representative images western blot images of TLR9, NLRP3, and cGAS protein expression in primary EVTs co-cultured with KYN-pretreated dMφs or control dMφs (n = 3 per group). (**E**) Representative immunofluorescence images showing co-localization of HLA-G and cGAS in hTSCs-EVTs co-cultured with KYN-pretreated dMφs or control dMφs (n = 5 per group). (**F**) Flow cytometry analysis of apoptosis levels in primary EVTs following co-culture with dMφs (n = 3 per group). (**G**) Scratch wound healing assay evaluating the migration capacity of HTR-8/SVneo cells (n = 3 per group). (**H**) Transwell invasion assay measuring the invasive ability of HTR-8/SVneo cells (n = 3 per group). (**I**) Western blot analysis of proteins associated with NF-κB signaling (p-NF-κB p65, NF-κB p65, p-IκBα, IκBα), apoptosis (BCL2, cleaved Caspase-3), and extracellular matrix remodeling (MMP9, MMP2) in HTR-8/SVneo cells. Data are presented as mean ± SD. Statistical significance was determined using the Student's t-test for two-group comparisons and one-way ANOVA for multiple comparisons; ns: not significant, *p < 0.05, **p < 0.01, ***p < 0.001.

A balanced immune microenvironment at the maternal-fetal interface is essential for successful pregnancy. Our scRNA-seq analysis revealed that *CGAS*, *NLRP3*, and *TLR9* were predominantly expressed in immune cells, with notably higher activation levels observed in macrophages, T cells, and plasma cells in the decidua of URPL pregnancies (Figure 6A). We also found that EVTs and endothelial cells derived from URPL pregnancies exhibited upregulation of *CGAS*, *NLRP3*, and *TLR9* (Figure 6A). These cells showed a transcriptomic signature indicative of inflammatory activation in the context of URPL (Figure 6B and Figure S10A-C). This coordinated upregulation of DNA-recognition receptors suggested that extracellular DNA may serve as an upstream inflammatory trigger in the URPL microenvironment.

Previous studies have suggested that extracellular mtDNA, released from autophagy-deficient cells, can modulate the behavior of neighboring cell types within the microenvironment 38, 39. Based on this, we hypothesized that, in addition to cytokine-mediated crosstalk, macrophages may also directly affect EVTs function through the release of mtDNA. To test this hypothesis, we cocultured primary EVTs, hTSCs-EVTs, and HTR-8/SVneo cells with either dMφs or THP-1-Mφs (Figure 6C). OE-AHR THP-1-Mφs markedly increased cGAS, NLRP3, and TLR9 expression in HTR-8/SVneo cells (Figure S11A). KYN-dMφs similarly elevated these sensors in primary EVTs (Figure 6D) and activated cGAS signaling in hTSCs-EVTs (Figure 6E). Purified macrophage-derived mtDNA induced a dose-dependent activation of the same pathway (Figure S11B). Functionally, KYN-dMφs triggered apoptosis (Figure 6F) and sharply reduced EVTs invasiveness (Figure S11C). HTR-8/SVneo cells exposed to OE-AHR THP-1-Mφs also showed increased apoptosis (Figure S11D) and diminished migration and invasion (Figure 6G, H). Notably, these effects were fully reversed by AHR inhibition with CH223191 and were partially alleviated by the STING inhibitor H-151 (Figure S12A-C), indicating that AHR-dependent macrophage mtDNA release, via cGAS-STING pathway, underlies EVTs dysfunction. Given that cGAS, TLR9, and NLRP3 commonly converge on NF-κB signaling 40-43, we next asked whether NF-κB pathway is activated in trophoblasts under these co-culture conditions. Interestingly, co-culture with OE-AHR THP-1-Mφs enhanced NF-κB activation in trophoblasts, as evidenced by increased phosphorylation of p65 and IκBα (Figure 6I). This effect was abolished following DNase I pre-treatment, confirming the involvement of extracellular DNA in the process (Figure 6I). Functionally, exposure to mtDNA or OE-AHR THP-1-Mφs led to downregulation of BCL2, upregulation of cleaved caspase-3, and suppression of MMP2/MMP9 expression (Figure 6I), collectively impairing trophoblast survival and invasiveness. Inhibition of NF-κB with JSH-23 reduced apoptosis and partially restored cell migration and invasion (Figure S13A-C), supporting a direct role for the mtDNA-NF-κB axis in mediating trophoblast dysfunction. Taken together, these findings indicate that CISH-induced degradation of ATP6V1A not only activates endogenous cGAS-STING signaling pathway via mtDNA but also affects the function of trophoblast cells by mtDNA release, resulting in pregnancy loss.

### CH223191 treatment alleviates embryo loss in an AP mouse model

**Figure 7:**
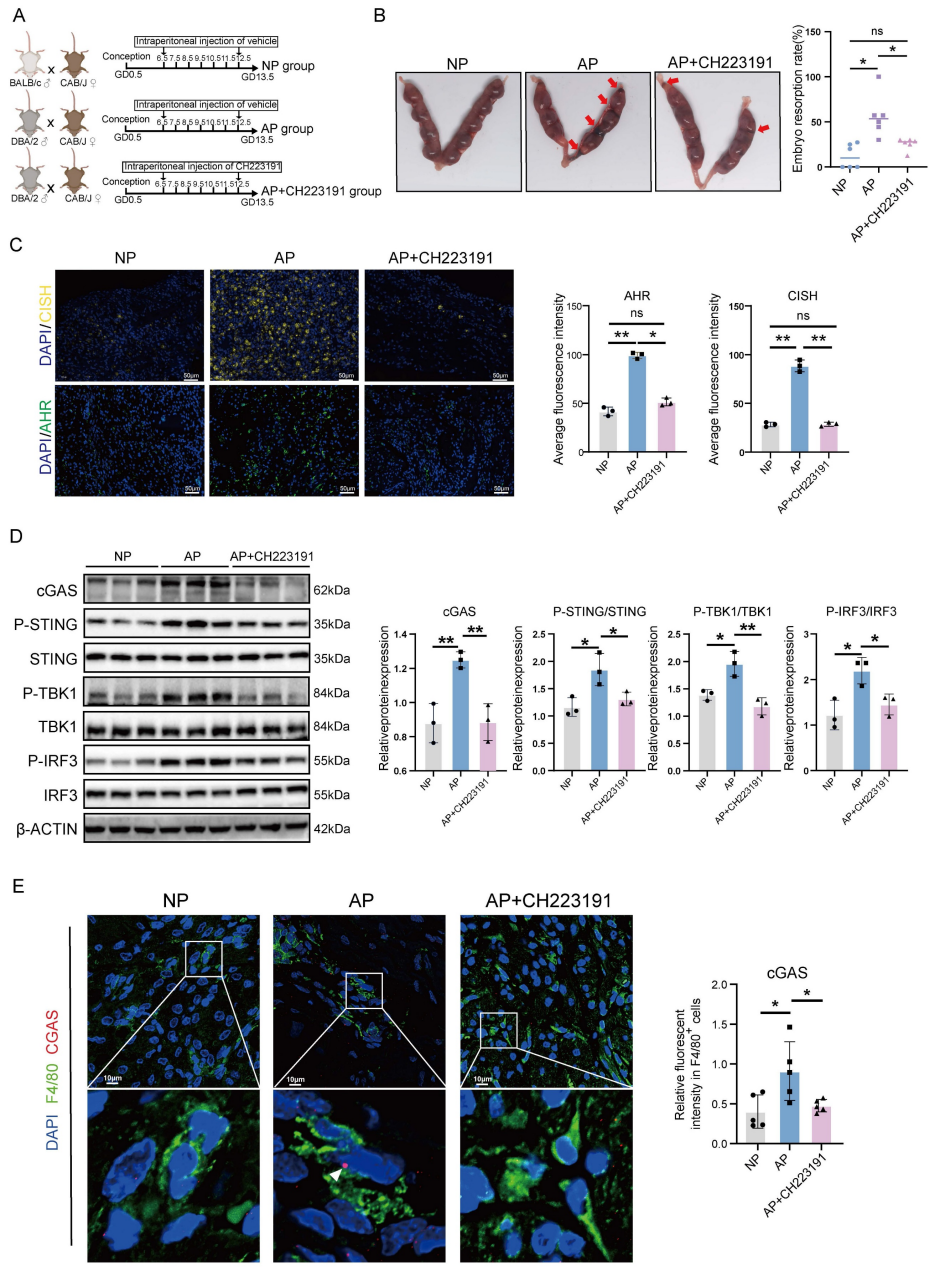
**AHR-activated macrophages promote embryo resorption in an abortion-prone mouse model.** (**A**) Schematic of the *in vivo* mouse models. CBA/J female mice were mated with BALB/c males to generate an NP model, or with DBA/2 males to establish an AP model. AP mice received daily intraperitoneal injections of CH223191 (20 μg/kg) or vehicle from GD 6.5 to GD 12.5. All mice were euthanized on GD 13.5. (**B**) Representative uterine images with resorbed embryos marked by red arrows. Total embryo resorption rates were quantified (n = 6 per group). (**C**) Representative immunofluorescence images showing AHR and CISH expression in decidual tissues from NP, AP, and AP mice treated with CH223191 (n = 3 per group). (**D**) Western blot and quantitative analysis of key components of the cGAS-STING signaling pathway, including cGAS, p-STING, STING, p-TBK1, TBK1, p-IRF3, and IRF3 protein levels in mouse decidual tissues from NP, AP, and AP mice treated CH223191 (n = 3 per group). (**E**) Representative immunofluorescence images showing co-localization of F4/80 and cGAS in mouse decidual tissues from NP and AP mice (n = 6 per group). Data are presented as mean ± SD. Statistical significance was assessed using the Student's t-test for two-group comparisons and one-way ANOVA for multiple-group comparisons; ns: not significant, *p < 0.01, **p < 0.01.

**Figure 8:**
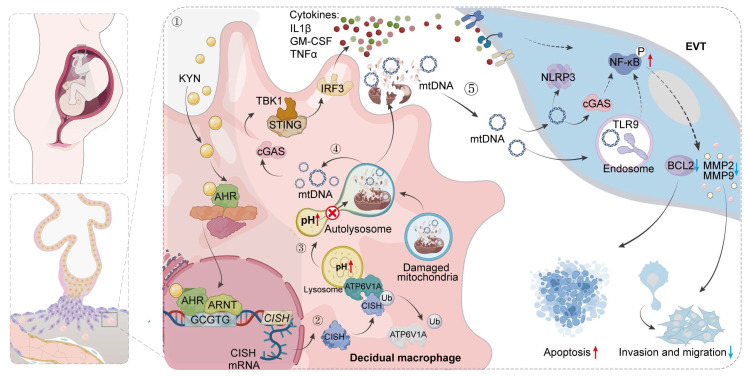
** Schematic illustration showing the mechanisms by which AHR^+^ dMφs regulate maternal-fetal tolerance in URPL pregnancies.** ① In URPL pregnancies, excessive levels of KYN in dMφs activate AHR-mediated transcription of CISH. ② Elevated CISH levels promote the ubiquitination and proteasomal degradation of ATP6V1A, a key subunit of the lysosomal V-ATPase complex responsible for maintaining lysosomal acidity. Loss of ATP6V1A impairs lysosomal acidification, resulting in lysosomal dysfunction. ③ This dysfunction facilitates the leakage of mtDNA into the cytosol and extracellular space. ④ Cytosolic mtDNA triggers the activation of cGAS-STING pathway, driving proinflammatory signaling and cytokine secretion from dMφs. ⑤ In parallel, extracellular mtDNA compromises trophoblast survival, migration, and invasion, thereby disrupting the immune-trophoblast interactions essential for maintaining decidual homeostasis. Collectively, the KYN-AHR-CISH-ATP6V1A axis mediates lysosomal and mitochondrial dysfunction in dMφs, contributing to a proinflammatory decidual microenvironment that may underlie the pathogenesis of URPL

After verifying that the AHR/CISH/ATP6V1A axis could promote immunological activation of macrophages and impair trophoblast function, we investigated the effect of CH223191, an inhibitor of AHR, on pregnancy maintenance *in vivo*. We established NP and AP mouse models, with a subset of AP mice receiving CH223191 to inhibit decidual AHR activity (Figure 7A). Notably, CH223191 treatment significantly reduced the embryo resorption rate observed in the AP group (Figure 7B). As shown in Figure 7C, CH223191 administration suppressed the expression of AHR and CISH in the decidua. We next assessed the activation of the cGAS-STING pathway. We found significant activation of the cGAS-STING pathway in AP mice, as evidenced by elevated cGAS expression and increased phosphorylation of downstream effectors, including STING, TBK1, and IRF3 (Figure 7D, E). Importantly, CH223191 treatment attenuated cGAS-STING activation. Immunofluorescence staining further revealed that cGAS expression was markedly reduced in dMφs following AHR blockade (Figure 7E), suggesting that AHR activity modulates the cGAS-STING pathway in dMφs in the AP mice. These findings indicate that CH223191 treatment alleviates embryonic loss and attenuates the activation of the cGAS-STING pathway in dMφs in the AP mice model. Taken together, our findings of associations between excessive kynurenine metabolism, impaired lysosomal acidification and mtDNA release in decidual macrophage from URPL pregnancies, indicate that abnormal dMφs kynurenine metabolism is an important contributor to the pathogenesis of URPL (Figure 8).

## Discussion

Dysregulation of the decidual immune microenvironment is a key contributor to the pathogenesis of URPL. Previous studies have demonstrated a significant increase in the number of dMφs in URPL pregnancies, accompanied by a shift toward a pro-inflammatory phenotype 44, which disrupts maternal-fetal tolerance and contributes to pregnancy failure. However, the involvement of tryptophan metabolism in dMφs and its relevance to URPL remain elusive. Employing multiple approaches in this study, we identified aberrant tryptophan metabolism in dMφs from URPL pregnancies, which was characterized by a significant increase in KYN. Mechanistically, KYN activates AHR, leading to mtDNA release and cGAS-STING pathway activation, which amplifies local inflammation. We further showed that AHR directly upregulates CISH, promoting ATP6V1A degradation and impairing lysosomal acidification, thereby facilitating mtDNA release. Moreover, AHR inhibition with CH223191 reduced fetal resorption *in vivo*, suggesting this pathway as a potential therapeutic target in URPL pregnancies. These results highlight the pivotal role of KYN in the regulation of CISH and underscore the importance of this regulatory axis in maintaining lysosomal homeostasis and immune balance during early pregnancy.

dMφs serve as pivotal immune regulators at the maternal-fetal interface, where they orchestrate a delicate balance between controlled pro-inflammatory responses and immunotolerance to ensure proper placental development 45. In this study, we identified dMφs as the most profoundly dysregulated immune subset in decidual tissues from URPL pregnancies, exhibiting a distinct pathological shift toward pro-inflammatory activation. Specifically, URPL-associated dMφs demonstrated markedly elevated secretion of TNF-α and IL-1β, aligning with previous observations of inflammatory dysregulation at the maternal-fetal interface in pregnancy loss 44, 46. The increased production of these pro-inflammatory cytokines is clinically significant, as they are likely key mediators of fetal rejection through multiple mechanisms 47-49. It has been reported that transient pro-inflammatory activation in dMφs is essential for trophoblast invasion, while persistent inflammation disrupts vascular remodeling and triggers trophoblast apoptosis 50. Furthermore, dysregulated dMφs impair regulatory T cell function, enhance dNK cell cytotoxicity, and disrupt immune equilibrium 51, 52. Supporting these findings, murine models underscore the criticality of balanced macrophage activity, as both depletion and hyperactivation precipitate pregnancy failure 13, 53. Thus, precise inflammatory regulation by dMφs is indispensable for maintaining gestational success.

Macrophage activation is intricately linked to metabolic reprogramming in response to microenvironmental cues 54. While classical immunometabolism paradigms associate glycolysis with pro-inflammatory activation, and fatty acid oxidation (FAO)/OXPHOS with anti-inflammatory states 55, 56, emerging research reveals a more complex, bidirectional regulation. It has been reported that glycolysis is essential for both pro-inflammatory responses and IL-4-mediated alternative activation, while FAO paradoxically contributes to NLRP3 inflammasome activation via mitochondrial ROS 57. This metabolic plasticity is further complicated by *in vivo* nutrient competition and tissue-specific demands. Yan *et al.* demonstrated that glycolytic flux promotes the pro-inflammatory polarization of dMφs in URPL pregnancies 58. In parallel, CD36-mediated lipid accumulation has been shown to further amplify inflammatory responses 16. Our work extends these findings by revealing enhanced amino acid, lipid, and glycolytic metabolism in URPL-associated dMφs, coupled with distinctive tryptophan catabolism dysregulation. Targeted metabolomics identified significant KYN pathway upregulation and serotonin suppression in URPL decidua. Given serotonin's critical role in placental vasodilation 59, its deficiency may impair fetoplacental circulation. Furthermore, as an endogenous ligand of the AHR, KYN is known to modulate immune responses. It is reported that elevated KYN/tryptophan ratios in cancer patients correlate with increased levels of IL-6, soluble IL-2 and expression of TNF-α receptors, and macrophage activation markers 60. Intriguingly, our data show that KYN treatment elevated the secretion of inflammatory cytokines (IL-1β, TNF-α) and chemokines (CCL3, IL-8) alongside a modest upregulation of the anti-inflammatory IL-10 in macrophages. Functionally, KYN-AHR-mediated secretome reprogramming of the dMφs may perturb immune homeostasis at the maternal-fetal interface. Elevated IL-1β and TNF-α levels are known to potentiate the cytotoxicity of NK cells through the upregulation of activating receptors such as NKp44 and NKG2D 61, while simultaneously suppressing FOXP3⁺ inducible regulatory T (iTreg) cell differentiation through the HIF-1α/mTORC1 and Akt/Smad3 signaling pathways, respectively 62, 63. Moreover, Huang *et al.* reported that IL-8 can induce trophoblasts to release TNF-α and IL-1β 64, thereby amplifying local pro-inflammatory cascades. Notably, both *in vitro* and *in vivo* studies have linked aberrant upregulation of IL-10 in the decidua with an increased risk of miscarriage 8, 65. Supporting these mechanisms, our *in vivo* experiments revealed that exposure to KYN in early pregnancy increased embryo resorption rates in normal pregnant mice, while administering the AHR inhibitor CH223191 to AP mice alleviated the embryo resorption rates. Our findings provide insight into the complex relationship between metabolic and immune processes in pregnancy and suggest that further exploration of the KYN-AHR axis could help refine therapeutic strategies for managing pregnancy complications and inflammatory conditions.

Emerging evidence highlights mitochondria as master regulators of cellular homeostasis, orchestrating not only energy metabolism and autophagy but also serving as critical signaling hubs for innate immune responses 66, 67. Recent studies have demonstrated that macrophage activation states are regulated by dynamic alterations in mitochondrial metabolism and physiology, including oxidative metabolism, membrane potential, TCA cycle, mtROS production, and mtDNA release 68. Zhang *et al.* showed that AHR activation, through crosstalk with the STAT3 signaling pathway, triggered a burst of mtROS, driving macrophage polarization toward a pro-inflammatory phenotype and contributed to tissue inflammation 69. Consistent with this, our results suggest that excessive activation of AHR leads to the accumulation of mtROS in macrophages, which subsequently causes mitochondrial dysfunction, characterized by depolarized Δψm and ultrastructural abnormalities.

Mitochondrial stress in inflammatory macrophages leads to the release of damage-associated molecular patterns (DAMPs), such as mtDNA, which act as potent activators of innate immune responses 70. These DAMPs stimulate pattern recognition receptors, including cGAS, which detects cytosolic dsDNA and initiates downstream signaling cascades. For example, during herpesvirus infection, mitochondrial stress triggers the release of mtDNA into the cytosol, where it is sensed by cGAS, leading to activation of the STING-IRF3 pathway and subsequent induction of type I interferon responses 71. Under normal conditions, cytosolic mtDNA and damaged mitochondria are cleared by the autophagy-lysosome system 72. However, lysosomal dysfunction can impair autophagy and mtDNA degradation, leading to cytosolic mtDNA accumulation and its release into the extracellular space. A study by Jin *et al.* showed that defective lysosomal acidification redirects undigested mtDNA into amphisomes, promoting its extracellular release 34. Our research builds on these findings by revealing that KYN disrupts lysosomal function in dMφs through a novel AHR/CISH/ATP6V1A regulatory axis. KYN activates AHR, which binds to the XRE2 motif (-323 to -319 bp) in the *CISH* promoter to drive its transcription. CISH recruits the Elongin B/C-Cullin5 E3 ubiquitin ligase complex via its SOCS-box domain 73, targeting ATP6V1A for ubiquitination and degradation. This process disrupts lysosomal acidification, causing defective autophagy-dependent mtDNA clearance and subsequent cytosolic accumulation and extracellular release of undegraded mtDNA. However, beyond the lysosomal-autophagic clearance axis delineated above, alterations in mitochondrial membrane permeability and vesicle/secretory-autophagy-mediated routes are also recognized contributors to mtDNA release. Mitochondrial oxidative stress can oxidize matrix-resident mtDNA, and oxidized mtDNA (ox-mtDNA) has been reported to exit mitochondria through the coordinated opening of the mitochondrial permeability transition pore (mPTP) and voltage-dependent anion channel (VDAC) 74. In addition, progressive enlargement of BAX/BAK macropores during apoptosis can drive bulk extrusion of mtDNA, thereby activating cGAS and other pattern-recognition receptors 75. Vesicle-dependent export-including mitochondria-derived vesicles and exosome secretion- and LC3-dependent secretory autophagy have likewise been implicated in routing mtDNA from the cytosol to the extracellular space 76. This study primarily focuses on the lysosomal acidification impairment pathway mediated by the KYN/AHR/CISH/ATP6V1A axis and has not yet performed systematic validation of other potential mtDNA release pathways. Therefore, we cannot completely rule out the possible auxiliary roles of these pathways in the context of KYN-AHR activation, which merits further investigation in future studies.

Extracellular mtDNA plays a well-documented role in sterile inflammation across multiple pathological contexts. In cancer, mtDNA released by senescent cells activates the cGAS-STING-NF-κB pathway in myeloid-derived suppressor cells, fostering tumor progression 29. Similarly, in preeclampsia, trophoblast-derived mtDNA contributes to endothelial dysfunction and placental vascular pathology 77. In the decidua of URPL pregnancies, we observed significant upregulation of cytosolic DNA sensors in decidual myeloid cells, EVTs, and endothelial cells, suggesting that persistent mtDNA stimulation may place the maternal-fetal interface under sustained inflammatory pressure. Our coculture experiments further showed that KYN-AHR upregulation in dMφs reduced EVTs invasion and migration and increased apoptosis. These findings indicate that increased mtDNA release, along with the activation of DNA-sensing receptors and downstream NF-κB signaling, serves as a central driver of these EVTs functional impairments. Nevertheless, contributions from cytokine-mediated parallel pathways cannot be excluded. Previous studies have demonstrated that TNF-α activates MAPK signaling and increases EVTs secretion of chemokines such as CCL2 and CCL5, potentially promoting monocyte-macrophage recruitment to the decidua 78. GM-CSF regulates trophoblast differentiation through STAT5, whereas IL-6 family cytokines modulate trophoblast survival and function via STAT3 activation 79, 80. Thus, within the immunologically privileged maternal-fetal interface, the coordinated effects of macrophage-derived mtDNA accumulation and cytokine dysregulation may disrupt intercellular communication, impair EVTs function, and interfere with decidual vascular remodeling, thereby contributing to defective placentation and pregnancy loss.

There are some limitations in this study. First, the limited availability of human decidual tissue restricted the ability to account for other potential determinants of pregnancy outcomes, such as maternal age, ethnicity, and comorbidities, which may limit the generalizability of the present findings. Future studies would benefit from larger, more diverse cohorts and stratified analyses to strengthen the robustness and clinical relevance of the conclusions. Second, our targeted metabolomics were conducted on whole decidual tissue rather than purified dMφs, as the small number of dMφs obtainable from clinical biopsies and the metabolic alterations introduced during isolation precluded reliable cell-type-specific profiling. Future studies with optimized isolation strategies or single-cell-resolved metabolomics will be essential for defining dMφs-intrinsic metabolic signatures. Third, while this study demonstrated that KYN promotes cytosolic accumulation and extracellular release of mtDNA in dMφs, the precise mechanisms responsible for the export of mtDNA from the cytosol to the extracellular space remain unclear. Potential pathways such as exosomes release, secretory autophagy, or alternative mechanisms were not explored and warrant future investigation. Fourth, due to the lack of an ideal human decidual macrophage cell line model, our conclusions relied heavily on *in vitro* experiments using THP-1-Mφs and dMφs isolated from clinical samples, which may not fully capture the complexity of the *in vivo* decidual microenvironment. Fifth, *in vivo* validation was conducted using a pharmacological AHR inhibitor rather than a macrophage-specific AHR knockout model, limiting the ability to discern cell-type-specific effects, as the inhibitor may exert off-target effects on trophoblasts, stromal cells, endothelial cells, and other immune cells. Furthermore, long-term outcomes such as fetal development and postpartum recovery were not evaluated. Future studies incorporating macrophage-specific genetic models and longitudinal analyses of pregnancy outcomes will be essential to validate the link between elevated decidual KYN levels and the risk of miscarriage or other pregnancy-related complications.

Collectively, using an integrative approach that combined single-cell transcriptomics, multiplex analysis, metabolomics, *in vitro* co-culture systems, and an *in vivo* AP mouse model, our findings uncovered a key link between KYN metabolism, lysosomal and mitochondrial integrity in dMφs, and immune regulation during early pregnancy. We demonstrate that excessive KYN activates the AHR/CISH/ATP6V1A signaling axis, leading to impaired lysosomal acidification, mitochondrial dysfunction, and cytosolic and extracellular leakage of mtDNA. These events trigger the activation of the cGAS-STING innate immune pathway and induce immunological activation in dMφs. In parallel, extracellular mtDNA compromises trophoblast survival, migration, and invasion, together disturbing decidual homeostasis and causing embryo loss. These insights expand our understanding of the cellular and molecular basis of URPL and provide a foundation for the development of immune-metabolic interventions to improve reproductive outcomes.

## Supplementary Material

Supplementary figures and tables.

## Abbreviations

dMφs: decidual macrophages
RPL: recurrent pregnancy loss
AHR: aryl hydrocarbon receptor
KYN: kynurenine
AP: abortion-prone
mtDNA: mitochondrial DNA
CISH: cytokine inducible SH2 containing protein
EVTs: extravillous trophoblasts
5-HIAA: 5-hydroxyindoleacetic acid
NP: normal pregnancy
THP-1-Mφs: THP-1-derived macrophages
dsDNA: cytosolic double-stranded DNA
SOCS: the suppressor of cytokine signaling
XREs: xenobiotic response elements
FAO: fatty acid oxidation
DAMPs: damage associated molecular patterns

## References

[1] Bender Atik R, Christiansen OB, Elson J, Kolte AM, Lewis S, Middeldorp S (2023). ESHRE guideline: recurrent pregnancy loss: an update in 2022. Human reproduction open.

[2] Quenby S, Gallos ID, Dhillon-Smith RK, Podesek M, Stephenson MD, Fisher J (2021). Miscarriage matters: the epidemiological, physical, psychological, and economic costs of early pregnancy loss. Lancet (London, England).

[3] Alecsandru D, Klimczak AM, Garcia Velasco JA, Pirtea P, Franasiak JM (2021). Immunologic causes and thrombophilia in recurrent pregnancy loss. Fertility and sterility.

[4] Vento-Tormo R, Efremova M, Botting RA, Turco MY, Vento-Tormo M, Meyer KB (2018). Single-cell reconstruction of the early maternal-fetal interface in humans. Nature.

[5] Guo C, Cai P, Jin L, Sha Q, Yu Q, Zhang W (2021). Single-cell profiling of the human decidual immune microenvironment in patients with recurrent pregnancy loss. Cell discovery.

[6] Pennisi E (2018). Tamed immune reaction aids pregnancy. Science (New York, NY).

[7] Bao S, Chen Z, Qin D, Xu H, Deng X, Zhang R (2023). Single-cell profiling reveals mechanisms of uncontrolled inflammation and glycolysis in decidual stromal cell subtypes in recurrent miscarriage. Human reproduction (Oxford, England).

[8] Zhu J, Song G, Zhou X, Han TL, Yu X, Chen H (2022). CD39/CD73 Dysregulation of Adenosine Metabolism Increases Decidual Natural Killer Cell Cytotoxicity: Implications in Unexplained Recurrent Spontaneous Abortion. Frontiers in immunology.

[9] Wang XQ, Zhou WJ, Hou XX, Fu Q, Li DJ (2018). Trophoblast-derived CXCL16 induces M2 macrophage polarization that in turn inactivates NK cells at the maternal-fetal interface. Cellular & molecular immunology.

[10] Erlebacher A (2013). Immunology of the maternal-fetal interface. Annual review of immunology.

[11] Zhao QY, Li QH, Fu YY, Ren CE, Jiang AF, Meng YH (2022). Decidual macrophages in recurrent spontaneous abortion. Frontiers in immunology.

[12] Gao Y, Mi N, Zhang Y, Li X, Guan W, Bai C (2022). Uterine macrophages as treatment targets for therapy of premature rupture of membranes by modified ADSC-EVs through a circRNA/miRNA/NF-κB pathway. Journal of nanobiotechnology.

[13] Care AS, Diener KR, Jasper MJ, Brown HM, Ingman WV, Robertson SA (2013). Macrophages regulate corpus luteum development during embryo implantation in mice. The Journal of clinical investigation.

[14] Meng YH, Zhou WJ, Jin LP, Liu LB, Chang KK, Mei J (2017). RANKL-mediated harmonious dialogue between fetus and mother guarantees smooth gestation by inducing decidual M2 macrophage polarization. Cell death & disease.

[15] Gao L, Xu QH, Ma LN, Luo J, Muyayalo KP, Wang LL (2022). Trophoblast-derived Lactic Acid Orchestrates Decidual Macrophage Differentiation via SRC/LDHA Signaling in Early Pregnancy. International journal of biological sciences.

[16] Chen J, Yin T, Hu X, Chang L, Sang Y, Xu L (2024). CD36-mediated arachidonic acid influx from decidual stromal cells increases inflammatory macrophages in miscarriage. Cell reports.

[17] Wei P, Dong M, Bi Y, Chen S, Huang W, Li T (2022). Identification and validation of a signature based on macrophage cell marker genes to predict recurrent miscarriage by integrated analysis of single-cell and bulk RNA-sequencing. Frontiers in immunology.

[18] Du L, Deng W, Zeng S, Xu P, Huang L, Liang Y (2021). Single-cell transcriptome analysis reveals defective decidua stromal niche attributes to recurrent spontaneous abortion. Cell proliferation.

[19] Skinnider MA, Squair JW, Kathe C, Anderson MA, Gautier M, Matson KJE (2021). Cell type prioritization in single-cell data. Nature biotechnology.

[20] Wagner A, Wang C, Fessler J, DeTomaso D, Avila-Pacheco J, Kaminski J (2021). Metabolic modeling of single Th17 cells reveals regulators of autoimmunity. Cell.

[21] Zhen XX, Yang L, Gu Y, Yang Q, Gu WW, He YP (2021). MNSFβ Regulates TNFα Production by Interacting with RC3H1 in Human Macrophages, and Dysfunction of MNSFβ in Decidual Macrophages Is Associated With Recurrent Pregnancy Loss. Frontiers in immunology.

[22] Chanput W, Mes JJ, Wichers HJ (2014). THP-1 cell line: an in vitro cell model for immune modulation approach. International immunopharmacology.

[23] Chen X, Song QL, Wang JY, Ji R, Cao ML, Guo DY (2023). FKBP5 regulates trophoblast-macrophage crosstalk in recurrent spontaneous abortion through PI3K/AKT and NF-κB signaling pathways. Free radical biology & medicine.

[24] Zhou X, Xu Y, Ren S, Yang N, Sun Y, Yang Q (2023). Trophoblast PR-SET7 dysfunction induces viral mimicry response and necroptosis associated with recurrent miscarriage. Proceedings of the National Academy of Sciences of the United States of America.

[25] Tilburgs T, Crespo Â C, van der Zwan A, Rybalov B, Raj T, Stranger B (2015). Human HLA-G+ extravillous trophoblasts: Immune-activating cells that interact with decidual leukocytes. Proceedings of the National Academy of Sciences of the United States of America.

[26] Okae H, Toh H, Sato T, Hiura H, Takahashi S, Shirane K (2018). Derivation of Human Trophoblast Stem Cells. Cell stem cell.

[27] Liu Z, Tang Y, Zhang X, Pei J, Wang C, Liu H (2023). Crosstalk between Placental Trophoblast and Decidual Immune Cells in Recurrent Miscarriage. International journal of medical sciences.

[28] Dai F, Zhang Y, Deng Z, Zhang J, Wang R, Chen J (2024). IGF2BP3 participates in the pathogenesis of recurrent spontaneous abortion by regulating ferroptosis. Journal of reproductive immunology.

[29] Gutiérrez-Vázquez C, Quintana FJ (2018). Regulation of the Immune Response by the Aryl Hydrocarbon Receptor. Immunity.

[30] Victorelli S, Salmonowicz H, Chapman J, Martini H, Vizioli MG, Riley JS (2023). Apoptotic stress causes mtDNA release during senescence and drives the SASP. Nature.

[31] Panwar V, Singh A, Bhatt M, Tonk RK, Azizov S, Raza AS (2023). Multifaceted role of mTOR (mammalian target of rapamycin) signaling pathway in human health and disease. Signal transduction and targeted therapy.

[32] Sun P, Yoshizuka N, New L, Moser BA, Li Y, Liao R (2007). PRAK is essential for ras-induced senescence and tumor suppression. Cell.

[33] van Loo G, Bertrand MJM (2023). Death by TNF: a road to inflammation. Nature reviews Immunology.

[34] Jin J, Mu Y, Zhang H, Sturmlechner I, Wang C, Jadhav RR (2023). CISH impairs lysosomal function in activated T cells resulting in mitochondrial DNA release and inflammaging. Nature aging.

[35] Shoger KE, Cheemalavagu N, Cao YM, Michalides BA, Chaudhri VK, Cohen JA (2021). CISH attenuates homeostatic cytokine signaling to promote lung-specific macrophage programming and function. Science signaling.

[36] Mindell JA (2012). Lysosomal acidification mechanisms. Annual review of physiology.

[37] Queval CJ, Song OR, Carralot JP, Saliou JM, Bongiovanni A, Deloison G (2017). Mycobacterium tuberculosis Controls Phagosomal Acidification by Targeting CISH-Mediated Signaling. Cell reports.

[38] Liu Z, Wang M, Wang X, Bu Q, Wang Q, Su W (2022). XBP1 deficiency promotes hepatocyte pyroptosis by impairing mitophagy to activate mtDNA-cGAS-STING signaling in macrophages during acute liver injury. Redox biology.

[39] Lai P, Liu L, Bancaro N, Troiani M, Calì B, Li Y (2025). Mitochondrial DNA released by senescent tumor cells enhances PMN-MDSC-driven immunosuppression through the cGAS-STING pathway. Immunity.

[40] Balka KR, Louis C, Saunders TL, Smith AM, Calleja DJ, D'Silva DB (2020). TBK1 and IKKε Act Redundantly to Mediate STING-Induced NF-κB Responses in Myeloid Cells. Cell reports.

[41] Waseem M, Imtiaz A, Alexander A, Graham L, Contreras-Galindo R (2025). Crosstalk between oxidative stress, mitochondrial dysfunction, chromosome instability, and the activation of the cGAS-STING/IFN pathway in systemic sclerosis. Ageing research reviews.

[42] Song C, He L, Zhang J, Ma H, Yuan X, Hu G (2016). Fluorofenidone attenuates pulmonary inflammation and fibrosis via inhibiting the activation of NALP3 inflammasome and IL-1β/IL-1R1/MyD88/NF-κB pathway. Journal of cellular and molecular medicine.

[43] Chen J, Wang T, Li X, Gao L, Wang K, Cheng M (2024). DNA of neutrophil extracellular traps promote NF-κB-dependent autoimmunity via cGAS/TLR9 in chronic obstructive pulmonary disease. Signal transduction and targeted therapy.

[44] Wang L, Wang H, Luo J, Xie T, Mor G, Liao A (2022). Decorin promotes decidual M1-like macrophage polarization via mitochondrial dysfunction resulting in recurrent pregnancy loss. Theranostics.

[45] Wang H, He M, Hou Y, Chen S, Zhang X, Zhang M (2016). Role of decidual CD14(+) macrophages in the homeostasis of maternal-fetal interface and the differentiation capacity of the cells during pregnancy and parturition. Placenta.

[46] Tsao FY, Wu MY, Chang YL, Wu CT, Ho HN (2018). M1 macrophages decrease in the deciduae from normal pregnancies but not from spontaneous abortions or unexplained recurrent spontaneous abortions. Journal of the Formosan Medical Association = Taiwan yi zhi.

[47] Zhang X, Fang Z, Wang X (2025). Gaps in maternal-fetal interface rejection response: chronic histiocytic intervillositis. Frontiers in immunology.

[48] Alijotas-Reig J, Esteve-Valverde E, Ferrer-Oliveras R, Llurba E, Gris JM (2017). Tumor Necrosis Factor-Alpha and Pregnancy: Focus on Biologics. An Updated and Comprehensive Review. Clinical reviews in allergy & immunology.

[49] Huang SJ, Chen CP, Schatz F, Rahman M, Abrahams VM, Lockwood CJ (2008). Pre-eclampsia is associated with dendritic cell recruitment into the uterine decidua. The Journal of pathology.

[50] Buckley RJ, Whitley GS, Dumitriu IE, Cartwright JE (2016). Macrophage polarisation affects their regulation of trophoblast behaviour. Placenta.

[51] Hsu P, Santner-Nanan B, Dahlstrom JE, Fadia M, Chandra A, Peek M (2012). Altered decidual DC-SIGN+ antigen-presenting cells and impaired regulatory T-cell induction in preeclampsia. The American journal of pathology.

[52] Co EC, Gormley M, Kapidzic M, Rosen DB, Scott MA, Stolp HA (2013). Maternal decidual macrophages inhibit NK cell killing of invasive cytotrophoblasts during human pregnancy. Biology of reproduction.

[53] Aikawa S, Deng W, Liang X, Yuan J, Bartos A, Sun X (2020). Uterine deficiency of high-mobility group box-1 (HMGB1) protein causes implantation defects and adverse pregnancy outcomes. Cell death and differentiation.

[54] Li J, Diao B, Guo S, Huang X, Yang C, Feng Z (2017). VSIG4 inhibits proinflammatory macrophage activation by reprogramming mitochondrial pyruvate metabolism. Nature communications.

[55] Harris AJ, Mirchandani AS, Lynch RW, Murphy F, Delaney L, Small D (2019). IL4Rα Signaling Abrogates Hypoxic Neutrophil Survival and Limits Acute Lung Injury Responses In Vivo. American journal of respiratory and critical care medicine.

[56] Xu F, Guo M, Huang W, Feng L, Zhu J, Luo K (2020). Annexin A5 regulates hepatic macrophage polarization via directly targeting PKM2 and ameliorates NASH. Redox biology.

[57] Van den Bossche J, O'Neill LA, Menon D (2017). Macrophage Immunometabolism: Where Are We (Going)?. Trends in immunology.

[58] Yan S, Ding J, Wang Z, Zhang F, Li J, Zhang Y (2023). CTRP6 regulates M1 macrophage polarization via the PPAR-γ/NF-κB pathway and reprogramming glycolysis in recurrent spontaneous abortion. International immunopharmacology.

[59] Cruz MA, Gallardo V, Miguel P, Carrasco G, González C (1997). Serotonin-induced vasoconstriction is mediated by thromboxane release and action in the human fetal-placental circulation. Placenta.

[60] Sperner-Unterweger B, Neurauter G, Klieber M, Kurz K, Meraner V, Zeimet A (2011). Enhanced tryptophan degradation in patients with ovarian carcinoma correlates with several serum soluble immune activation markers. Immunobiology.

[61] Mattiola I, Pesant M, Tentorio PF, Molgora M, Marcenaro E, Lugli E (2015). Priming of Human Resting NK Cells by Autologous M1 Macrophages via the Engagement of IL-1β, IFN-β, and IL-15 Pathways. Journal of immunology (Baltimore, Md: 1950).

[62] Feldhoff LM, Rueda CM, Moreno-Fernandez ME, Sauer J, Jackson CM, Chougnet CA (2017). IL-1β induced HIF-1α inhibits the differentiation of human FOXP3(+) T cells. Scientific reports.

[63] Zhang Q, Cui F, Fang L, Hong J, Zheng B, Zhang JZ (2013). TNF-α impairs differentiation and function of TGF-β-induced Treg cells in autoimmune diseases through Akt and Smad3 signaling pathway. Journal of molecular cell biology.

[64] Huang Z, Du G, Huang X, Han L, Han X, Xu B (2018). The enhancer RNA lnc-SLC4A1-1 epigenetically regulates unexplained recurrent pregnancy loss (URPL) by activating CXCL8 and NF-kB pathway. EBioMedicine.

[65] Samudra AN, Dwyer KM, Selan C, Freddi S, Murray-Segal L, Nikpour M (2018). CD39 and CD73 activity are protective in a mouse model of antiphospholipid antibody-induced miscarriages. Journal of autoimmunity.

[66] Nunnari J, Suomalainen A (2012). Mitochondria: in sickness and in health. Cell.

[67] Li W, Cai J, Gu Y, Li X, He W, Chen Y (2025). Novel pH-responsive and CD44-targeting silica nanoparticles for inflammatory bowel disease therapy. Chemical Engineering Journal.

[68] Wang Y, Li N, Zhang X, Horng T (2021). Mitochondrial metabolism regulates macrophage biology. The Journal of biological chemistry.

[69] Zhang Q, Liu M, Zhang J, Jiang H, Ma C, Jian Y (2024). Macrophage MAPK7/AhR/STAT3 Signaling Mediates Mitochondrial ROS Burst and Enterohepatic Inflammatory Responses Induced by Deoxynivalenol Relevant to Low-Dose Exposure in Children. Environmental science & technology.

[70] Kerur N, Fukuda S, Banerjee D, Kim Y, Fu D, Apicella I (2018). cGAS drives noncanonical-inflammasome activation in age-related macular degeneration. Nature medicine.

[71] West AP, Khoury-Hanold W, Staron M, Tal MC, Pineda CM, Lang SM (2015). Mitochondrial DNA stress primes the antiviral innate immune response. Nature.

[72] Liu H, Zhen C, Xie J, Luo Z, Zeng L, Zhao G (2024). TFAM is an autophagy receptor that limits inflammation by binding to cytoplasmic mitochondrial DNA. Nature cell biology.

[73] Wang B, Wangkahart E, Secombes CJ, Wang T (2019). Insights into the Evolution of the Suppressors of Cytokine Signaling (SOCS) Gene Family in Vertebrates. Molecular biology and evolution.

[74] Xian H, Watari K, Sanchez-Lopez E, Offenberger J, Onyuru J, Sampath H (2022). Oxidized DNA fragments exit mitochondria via mPTP- and VDAC-dependent channels to activate NLRP3 inflammasome and interferon signaling. Immunity.

[75] Cosentino K, Hertlein V, Jenner A, Dellmann T, Gojkovic M, Peña-Blanco A (2022). The interplay between BAX and BAK tunes apoptotic pore growth to control mitochondrial-DNA-mediated inflammation. Molecular cell.

[76] Newman LE, Weiser Novak S, Rojas GR, Tadepalle N, Schiavon CR, Grotjahn DA (2024). Mitochondrial DNA replication stress triggers a pro-inflammatory endosomal pathway of nucleoid disposal. Nature cell biology.

[77] Lv Z, Lv DY, Meng JY, Sha XY, Qian XY, Chen YS (2023). Trophoblastic mitochondrial DNA induces endothelial dysfunction and NLRP3 inflammasome activation: Implications for preeclampsia. International immunopharmacology.

[78] Renaud SJ, Sullivan R, Graham CH (2009). Tumour necrosis factor alpha stimulates the production of monocyte chemoattractants by extravillous trophoblast cells via differential activation of MAPK pathways. Placenta.

[79] Robertson SA (2007). GM-CSF regulation of embryo development and pregnancy. Cytokine & growth factor reviews.

[80] Ravelojaona M, Girouard J, Kana Tsapi ES, Chambers M, Vaillancourt C, Van Themsche C (2024). Oncostatin M and STAT3 Signaling Pathways Support Human Trophoblast Differentiation by Inhibiting Inflammatory Stress in Response to IFNγ and GM-CSF. Cells.

