# Supplementary material for: Excessive Kynurenine Metabolism Impairs Lysosomal acidification and Triggers mtDNA Release via the AHR/CISH/ATP6V1A Axis in Decidual Macrophages Associated with Unexplained Recurrent Pregnancy Loss
Source: Int J Biol Sci. 2026 Jan 1;22(2):895–919. doi: 10.7150/ijbs.121947 (PMC12781176; doi:10.7150/ijbs.121947)
Supplement: Supplementary file 1 — Supplementary figures and tables. [file ijbsv22p0895s1.pdf]

Supporting Information

Excessive Kynurenine Metabolism Impairs Lysosomal acidification and Triggers mtDNA Release via the AHR/CISH/ATP6V1A Axis in Decidual Macrophages associated with Unexplained Recurrent Pregnancy Loss

Guangmin Song[1, †], Hongli Li[1, †], Man Zhang[1, †], Yun Li[1], Xinyi Tao[1], Andi Wang[1], Jianqi Wang[1], Boris Novakovic[2], Richard D. Cannon[3], Richard Saffery[2], Hongbo Qi[1], Hua Zhang[1], Xiaobo Zhou[1, *]

[1]Department of Obstetrics and Gynecology, Chongqing Key Laboratory of Maternal and Fetal Medicine / Joint International Research Laboratory of Reproduction & Development, Ministry of Education / The Innovation and Talent Recruitment Base of Maternal-Fetal Medicine, The First Affiliated Hospital of Chongqing Medical University, No.1 Youyi Rd, Yuzhong District, Chongqing, 400016, China.

[2]Molecular Immunity, Murdoch Children's Research Institute and Department of Paediatrics, University of Melbourne, Melbourne, VIC, Australia.

[3]Department of Oral Sciences, Sir John Walsh Research Institute, Faculty of Dentistry, University of Otago, Dunedin, New Zealand.

[†]Guangmin Song, Hongli Li and Man Zhang contributed equally to this work.

[*]To whom correspondence may be addressed. Email: xiaobo_zhou@cqmu.edu.cn (Xiaobo Zhou)

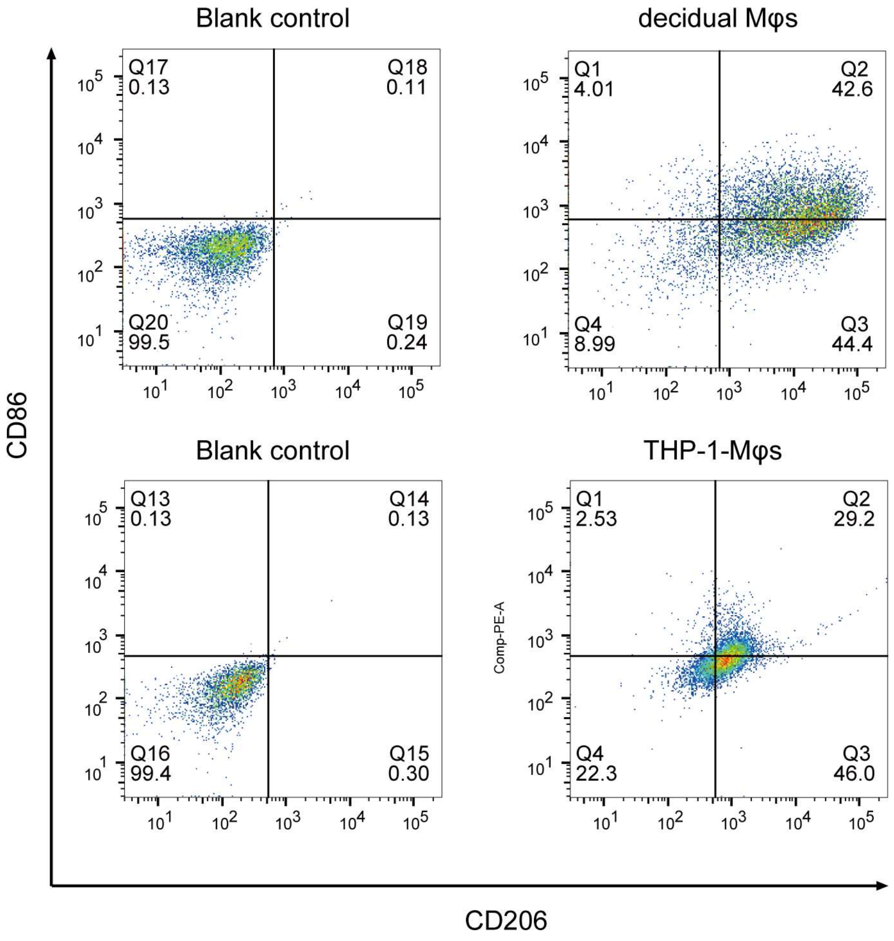

Figure S1. Flow cytometry analysis of surface markers (CD86, CD206) in primary decidual macrophages (dMφs) and THP-1–derived macrophages (THP-1-Mφs).

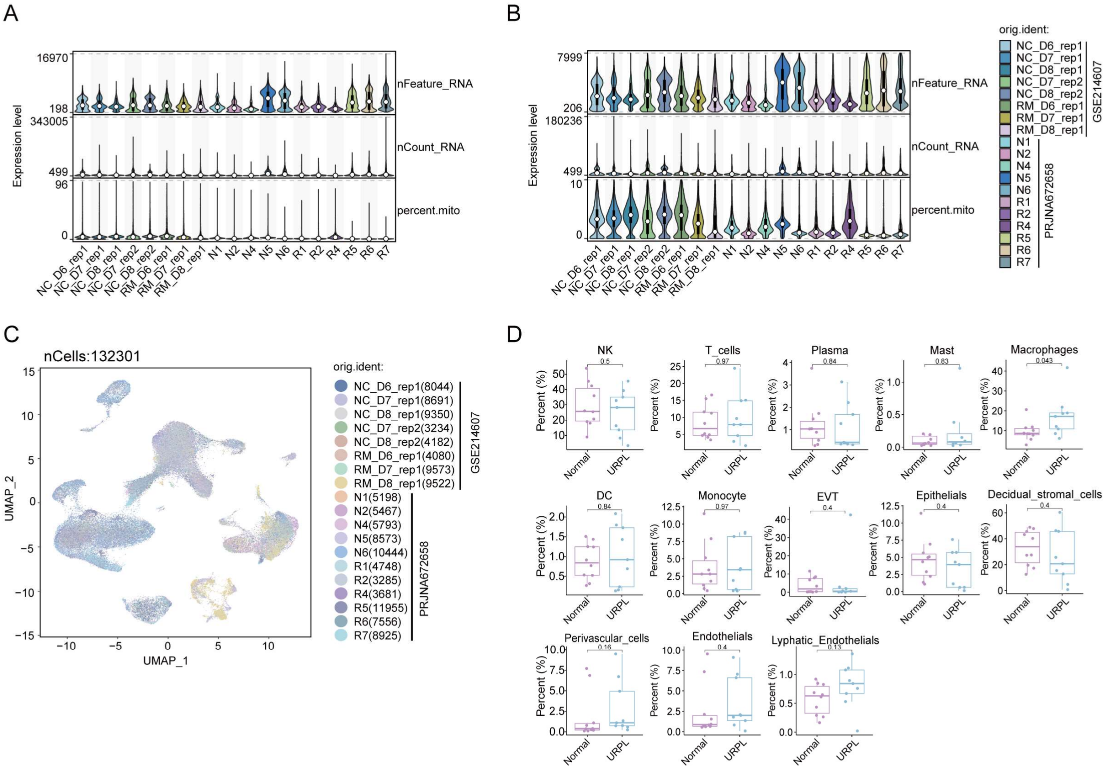

Figure S2. Quality control and cell type distribution of scRNA-seq data from decidual tissues. (A-B) Violin plots showing the distribution of the number of detected genes (nFeature_RNA), total RNA counts (nCount_RNA), and the percentage of mitochondrial genes (percent.mito) across cells from 19 decidual tissue samples before (A) and after (B) quality control filtering. (C) UMAP plot illustrating sample integration of individual samples, grouped by original identity (orig.ident). (D) Box plots displaying the relative proportion of each identified cell type within the total cell population across all decidual tissue samples.

.

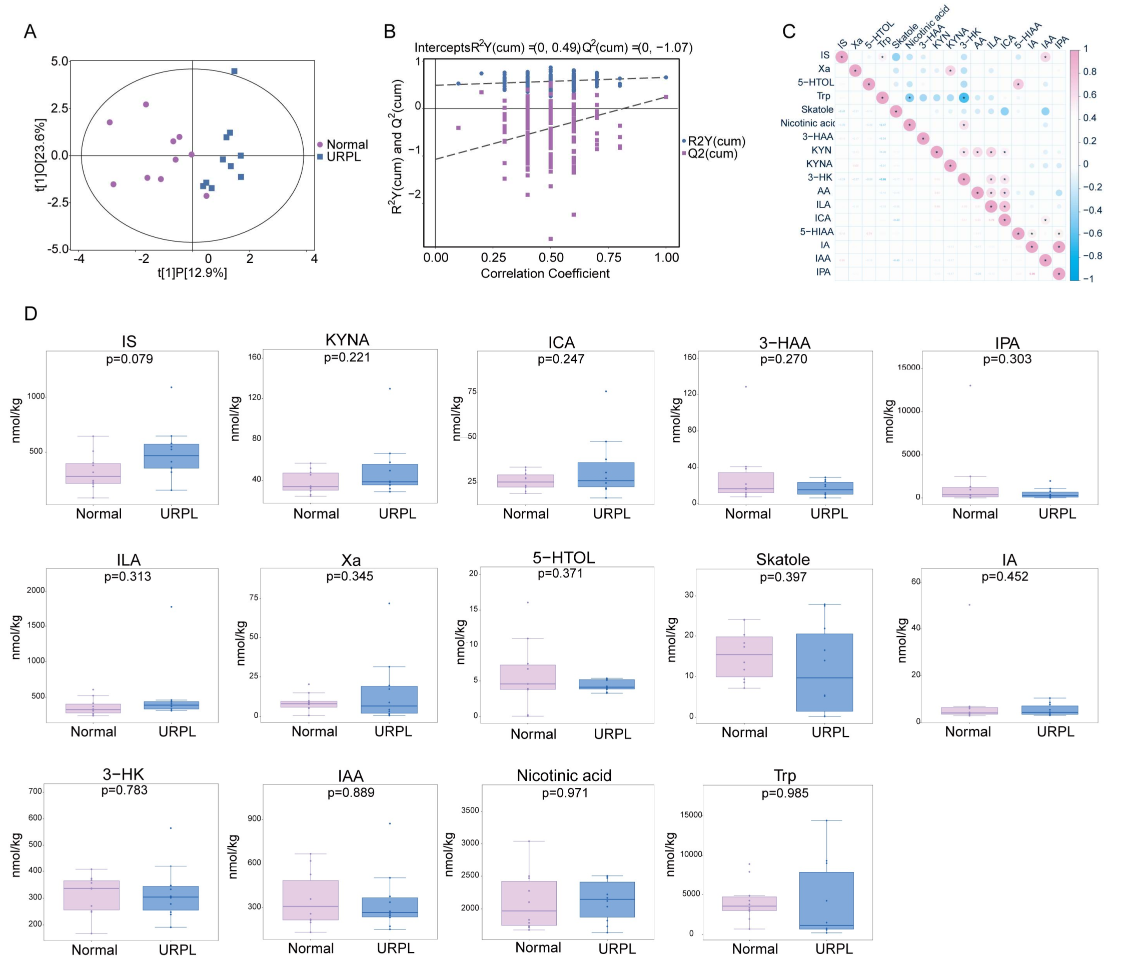

Figure S3. Metabolomic profiling of tryptophan metabolites in decidual tissues from URPL and normal pregnancies. (A) OPLS-DA score plot illustrating the separation of metabolic profiles between the decidual tissues from URPL and normal pregnancies. (B) OPLS-DA model evaluation plot demonstrating the model's goodness of fit (R^2Y) and predictive power (Q^2), alongside the distribution of permutation test results for model validation. (C) Correlation heatmap of the 17 identified metabolites. (D) Box plots comparing the concentrations of 14 tryptophan metabolites between the normal and URPL pregnancies (n = 10 per group). Statistical significance was determined using Student's t-test.

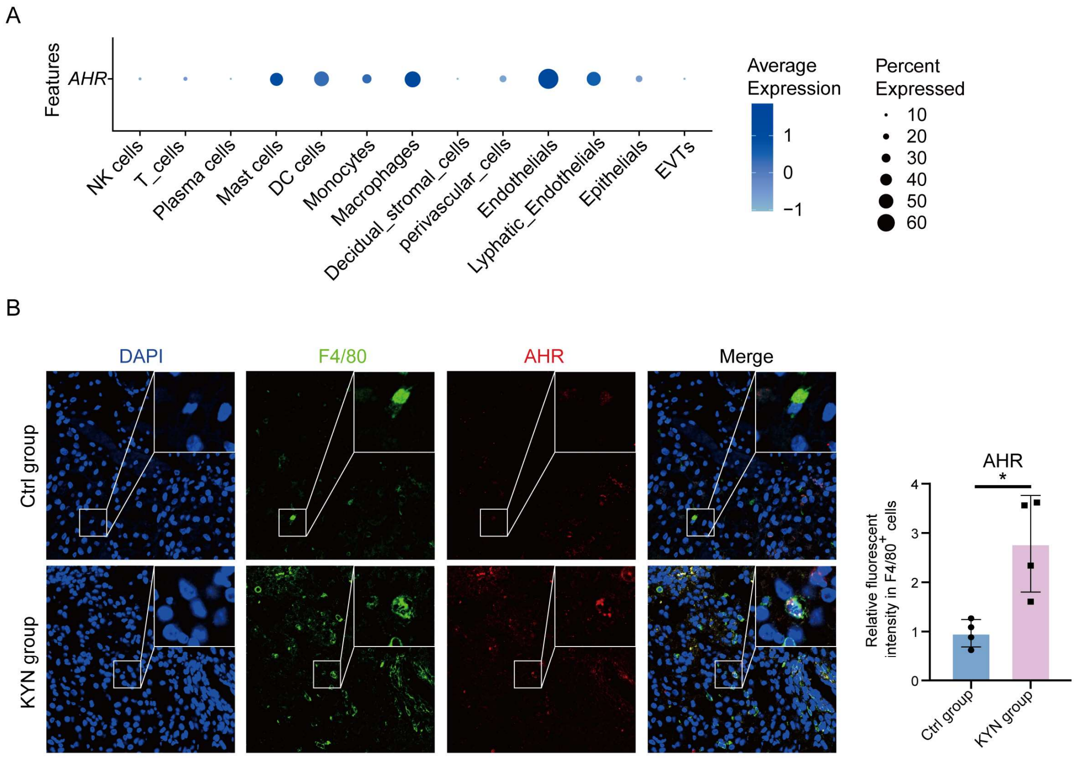

Figure S4. AHR expression in human decidual tissues and mouse decidual macrophages. (A) Dot plot illustrating the level of AHR expression across distinct decidual cell populations based on human scRNA-seq data. (B) Immunofluorescence images of AHR and F4/80 co-staining in the decidua from KYN- and PBS-treated mice. Data are presented as mean ± SD, n=5. Statistical significance was determined using the Student's t-test; **p* < 0.05.

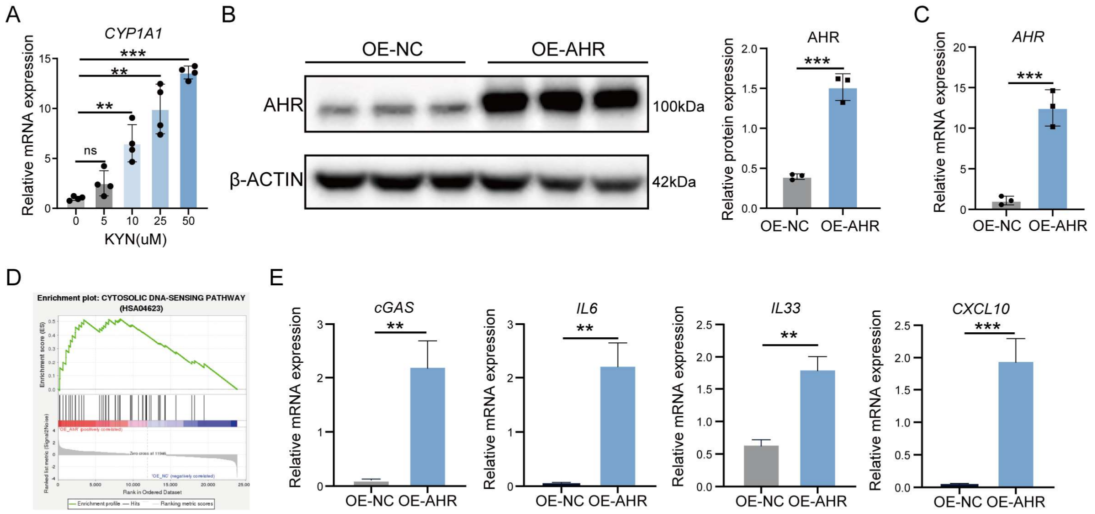

Figure S5. AHR overexpression activates cytosolic DNA sensing in THP-1-Mφs. (A) RT-qPCR analysis of CYP1A1 mRNA expression in THP-1-Mφs treated with increasing concentrations of KYN (0, 5, 10, 25, and 50 μM) (n = 4 per group). (B-C) Western blot (B) and RT-qPCR (C) analysis of AHR protein and mRNA expression in OE-AHR and OE-NC THP-1-Mφs (n = 4 per group). (D) GSEA plot highlighting enrichment of the cytosolic DNA sensing pathway in cells overexpressing AHR. (E) RT-qPCR analysis confirming the impact of AHR overexpression on the expression of *cGAS*, *IL6*, *IL33*, and *CXCL10* in THP-1-Mφs (n = 4 per group). Data are presented as mean ± SD. Statistical significance was assessed using the Student's t-test for two-group comparisons and one-way ANOVA for multiple comparisons; ns: not significant, $^{**}p < 0.01$, $^{***}p < 0.001$.

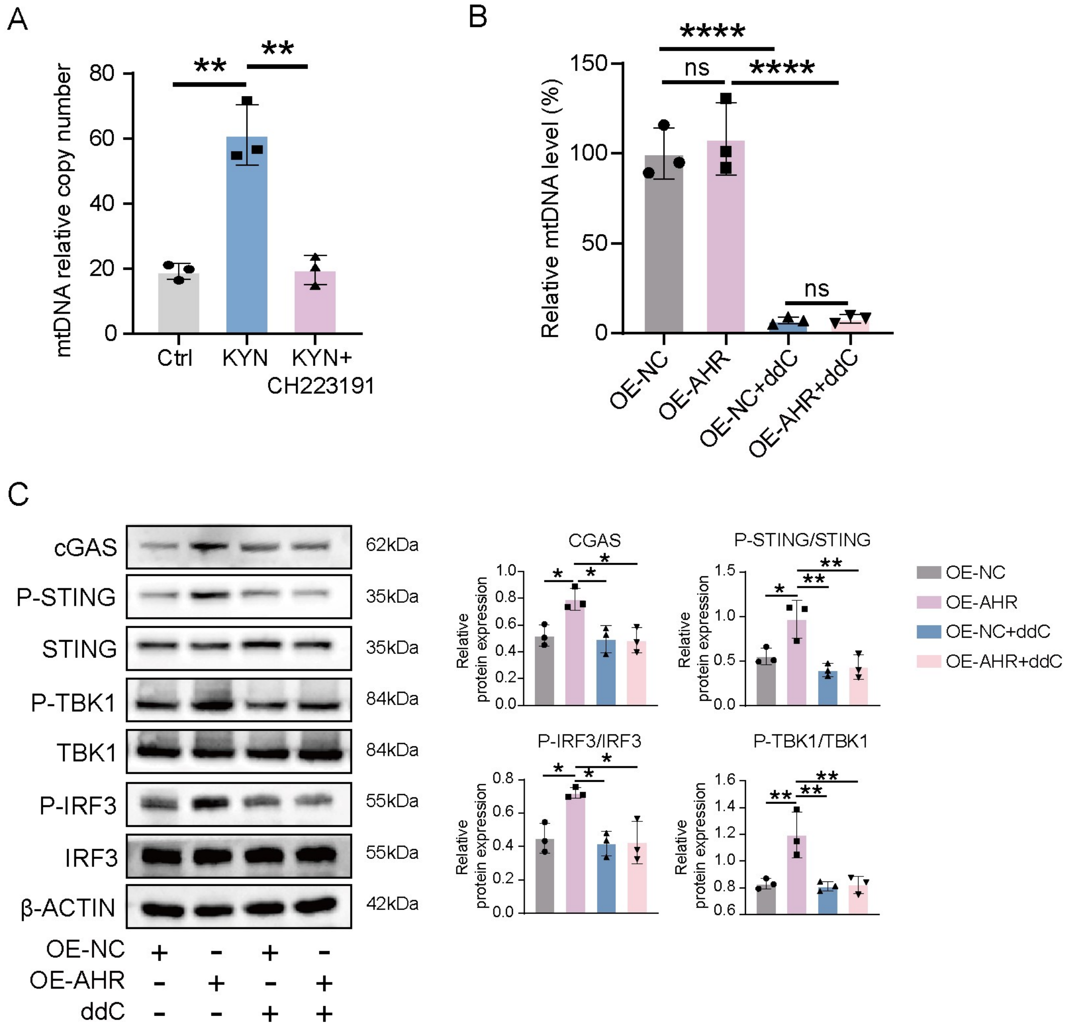

Figure S6. mtDNA-dependent cGAS-STING activation in macrophages overexpressing AHR. (A) THP-1-Mφs were cultured under control conditions or stimulated with KYN, with or without pretreatment with the AHR inhibitor CH223191. DNA was isolated from cell-free culture supernatants, and mtDNA copy number was quantified by RT-qPCR (n = 3 per group). (B) 2',3'-dideoxycytidine (ddC) or DMSO control were added to the culture medium of OE-NC or OE-AHR THP-1 cells for four days to block mtDNA replication. Total cellular mtDNA content was quantified as the mtDNA-to-nDNA ratio. (C) Western blot analysis for indicated proteins involved in the

cGAS–STING pathway, including cGAS, p-STING, STING, p-TBK1, TBK1, p-IRF3, and IRF3, in OE-AHR and OE-NC THP-1 cells untreated or treated with ddC ($n = 3$ per group). Data are presented as mean ± SD. Statistical significance was assessed using the Student's t-test; ns: not significant, *$p < 0.05$, **$p < 0.01$, ****$p < 0.0001$.

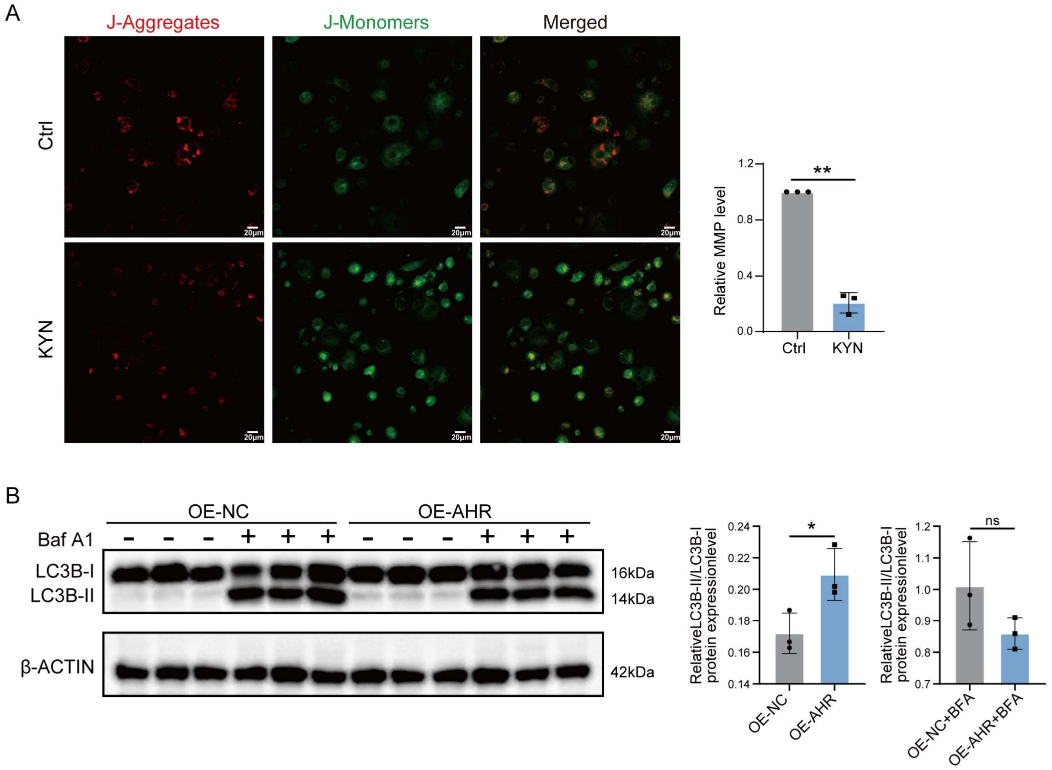

Figure S7. AHR activation impairs mitochondrial and lysosomal function in THP-1-Mφs. (A) JC-1 staining to evaluate mitochondrial membrane potential (ΔΨm) in KYN-treated and control THP-1-Mφs. Mitochondrial membrane potential was quantified by the red-to-green fluorescence ratio. (B) Western blot analysis of LC3B-I and LC3B-II protein levels in OE-AHR and OE-NC THP-1-Mφs treated with or without bafilomycin A1 (Baf A1, n = 3). Data are presented as mean ± SD. Statistical significance was assessed using the Student's t-test; ns: not significant, *$p < 0.05$, **$p < 0.01$.

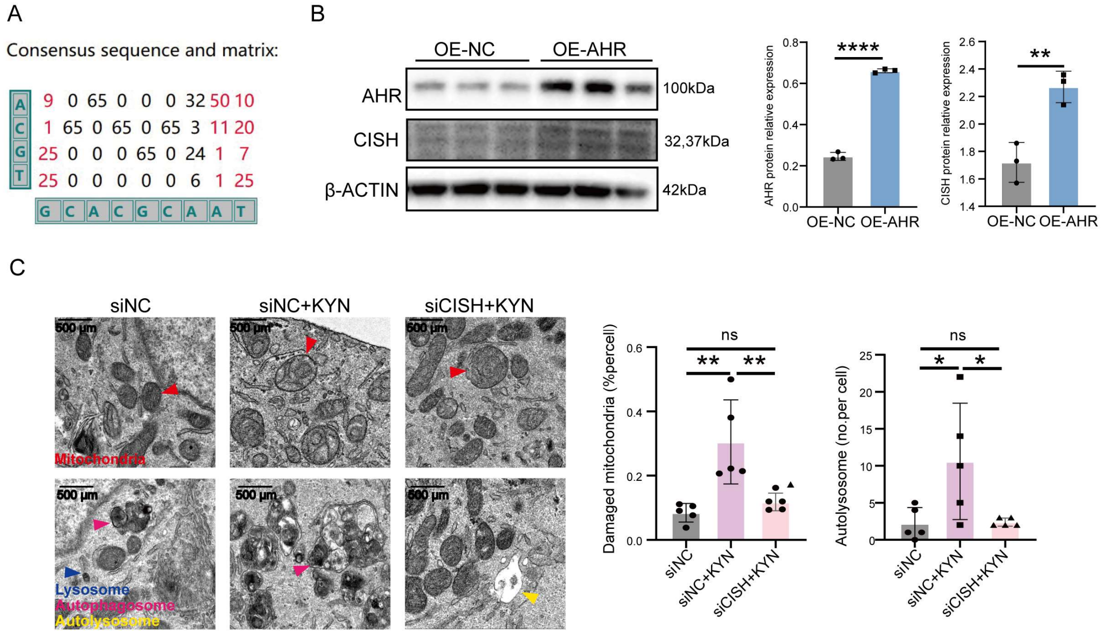

Figure S8. KYN regulates lysosomal function through CISH. (A) Predicted AHR-binding motif in the CISH promoter region based on the PROMO database. (B) Western blot analysis showing increased CISH protein expression in THP-1-Mφs overexpressing AHR (n = 3 per group). (C) TEM images showing the ultrastructural morphology of mitochondria, lysosomes, autophagosomes, and autolysosomes in each group of THP-1-Mφs (n = 5 per group). Data are presented as mean ± SD. Statistical significance was determined using the Student's t-test for two-group comparisons and one-way ANOVA for multiple comparisons; ns: not significant, *$p < 0.05$, **$p < 0.01$, ****$p < 0.0001$.

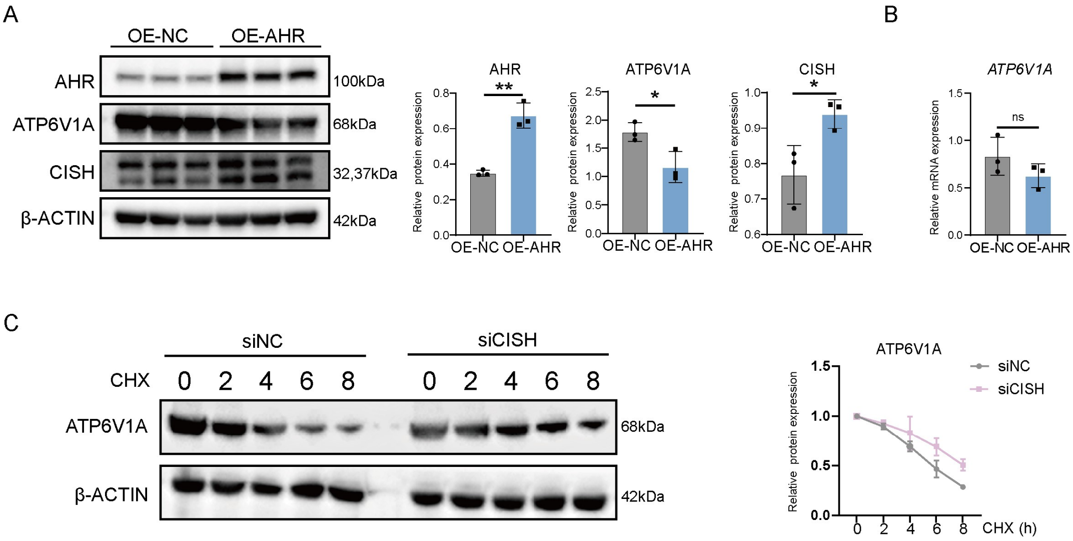

Figure S9. CISH regulates the protein stability of ATP6V1A. (A) Western blotting analysis of AHR, ATP6V1A, and CISH in OE-NC and OE-AHR THP-1-Mφs (n = 3 per group). (B) RT-qPCR analysis of *ATP6V1A* mRNA levels in OE-NC and OE-AHR THP-1-Mφs (n = 3 per group). (C) THP-1-Mφs were transfected with either negative control siRNA or CISH-specific siRNA, followed by treatment with cycloheximide (CHX, 10 μg/mL) for the indicated durations. ATP6V1A protein stability was evaluated by western blotting (n = 3 per group). Data are presented as mean ± SD. Statistical significance was determined using the Student's t-test; ns: not significant, $*p < 0.05$, $**p < 0.01$.

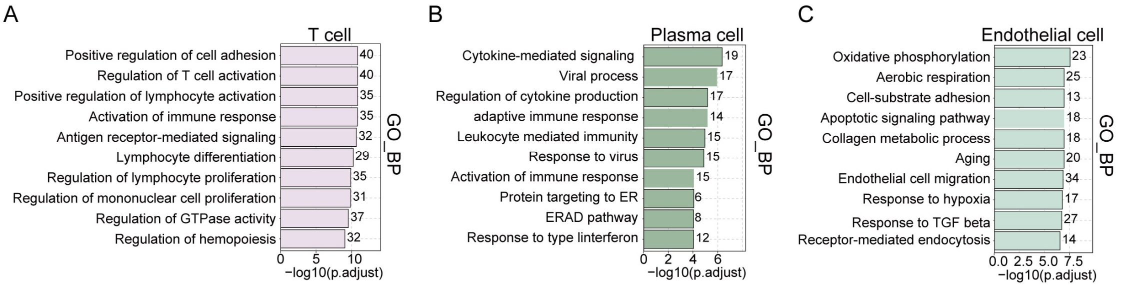

Figure S10. Significantly enriched GO terms in decidual T cells (A), plasma cells (B) and endothelial cells (C) from URPL pregnancies based on scRNA-seq data.

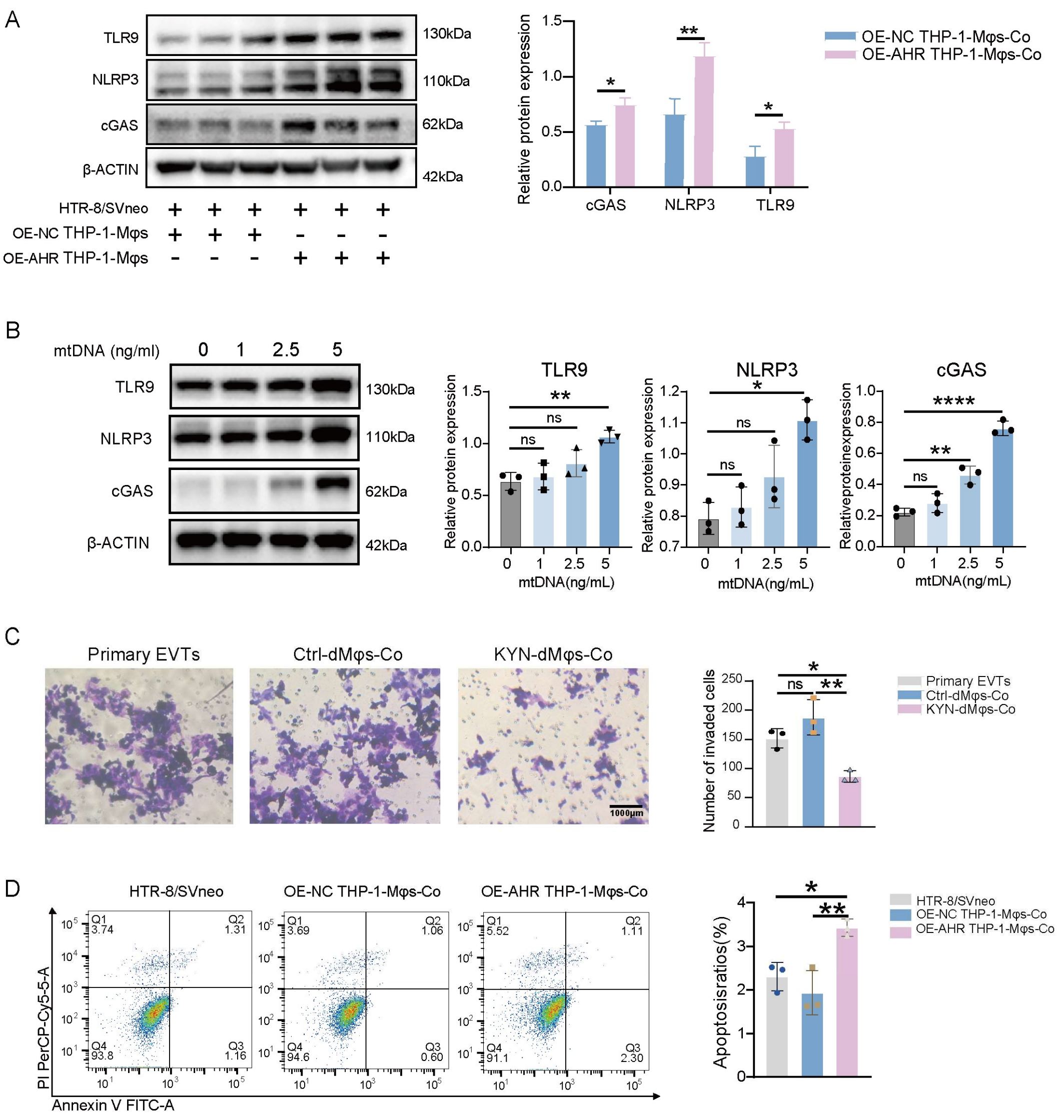

Figure S11. AHR-activated macrophages induce DNA-sensing pathways and impair EVTs function. (A) Representative western blot images of TLR9, NLRP3, and cGAS protein expression in HTR-8/SVneo cells co-cultured with OE-AHR or OE-NC THP-1-Mφs. (B) Protein expression levels of TLR9, NLRP3, and cGAS in HTR-8/SVneo cells treated with increasing concentrations of extracellular mtDNA (0, 1, 2.5, and 5 ng/mL) for 24 h. (C) Transwell invasion assay measuring the invasive ability of primary EVTs. (D) Flow cytometry analysis of apoptosis levels in HTR-8/SVneo cells following co-

culture with THP-1-Mφs. Data are presented as mean ± SD, n=3. Statistical significance was determined using the Student's t-test for two-group comparisons and one-way ANOVA for multiple comparisons; ns: not significant, *$p < 0.05$, **$p < 0.01$, ****$p < 0.0001$.

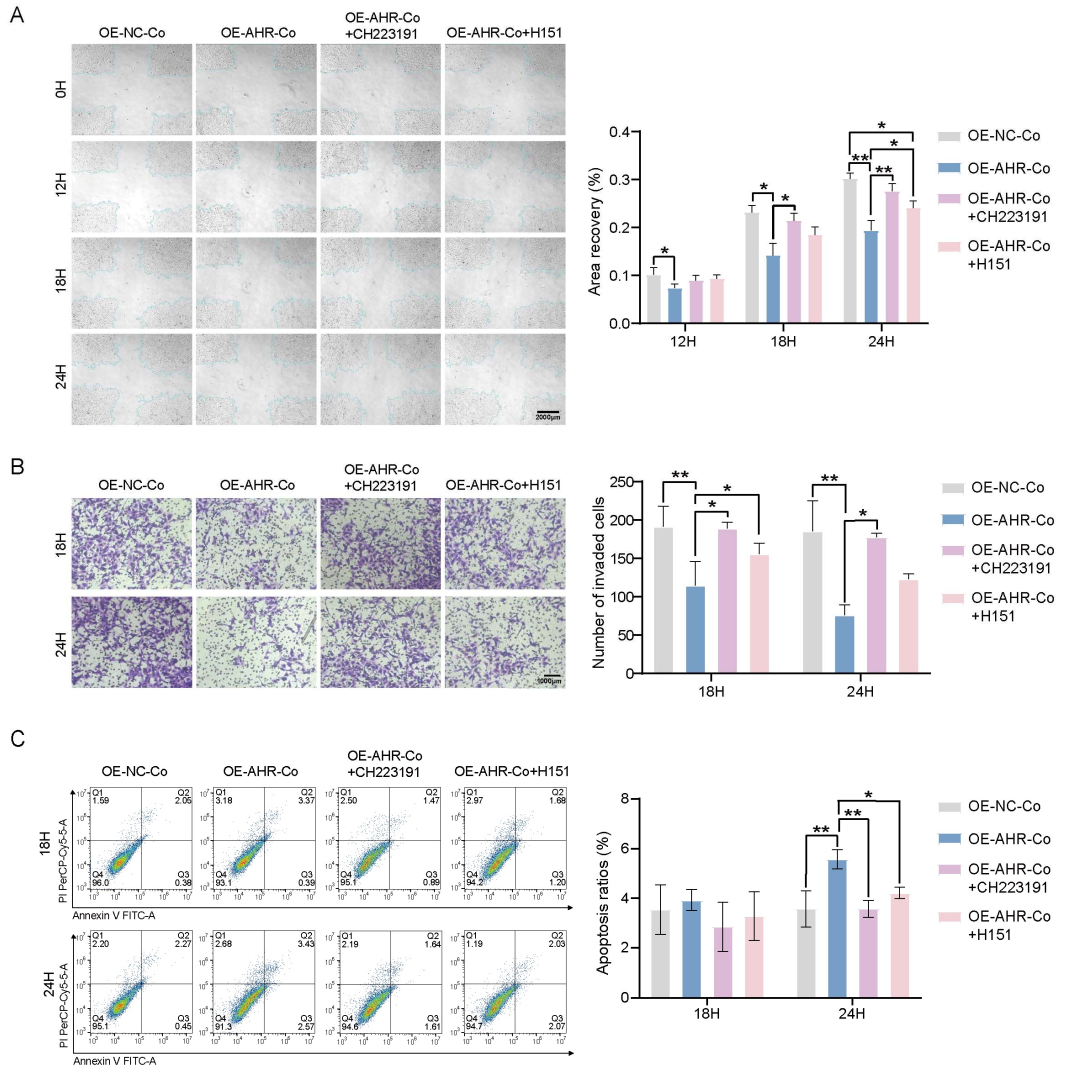

Figure S12. Effects of AHR and STING inhibition on macrophage-induced trophoblast dysfunction. (A-B) Scratch wound healing assay (A) and transwell assay (B) evaluating the migratory and invasive capacities of HTR-8/SVneo cells co-cultured with OE-NC THP-1-Mφs, OE-AHR THP-1-Mφs, OE-AHR THP-1-Mφs pretreated with CH223191, or OE-AHR THP-1-Mφs with H151. (C) Flow cytometry analysis of apoptosis levels in HTR-8/SVneo cells under the indicated conditions. Data are presented as mean ± SD, n=3. Statistical significance was determined by one-way ANOVA; ns: not significant, **p* < 0.05, ***p* < 0.01.

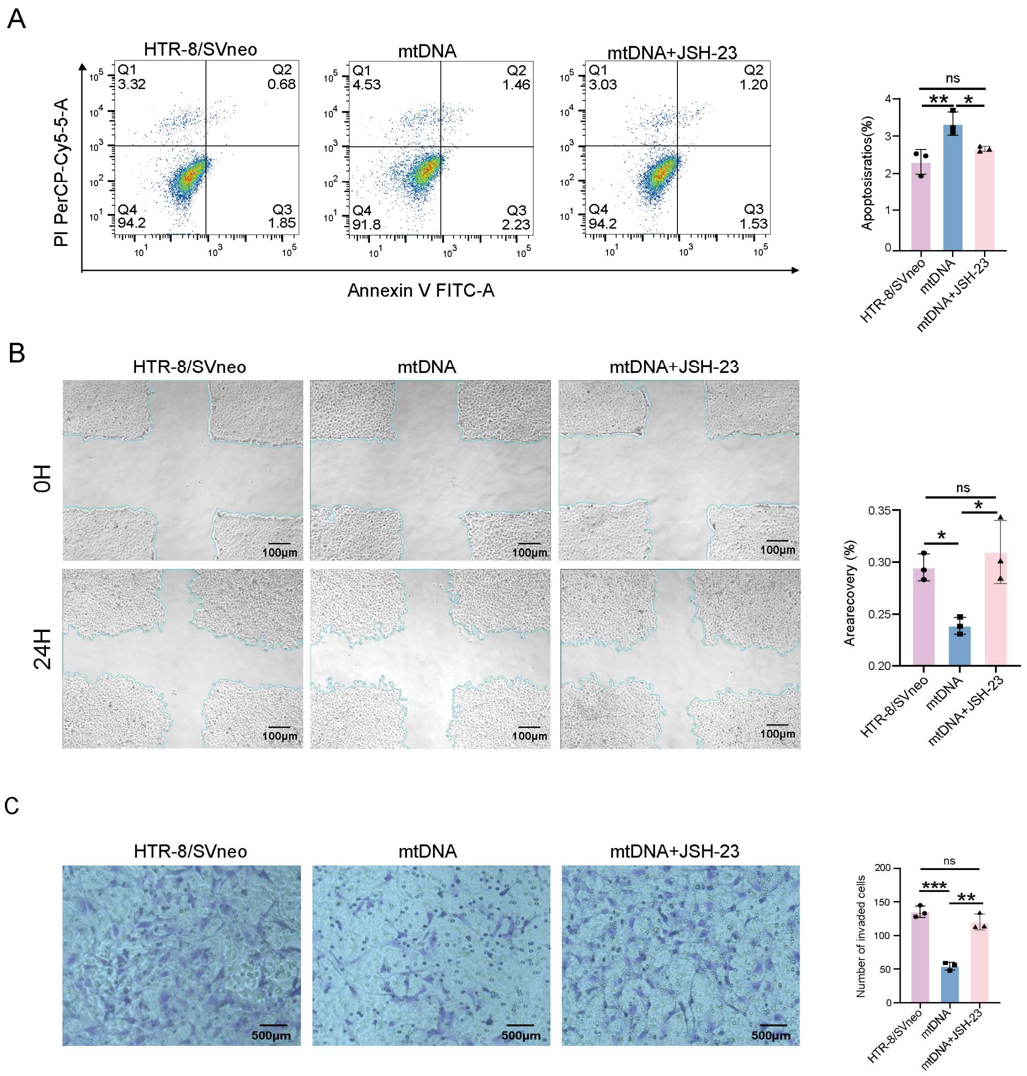

Figure S13. Extracellular mtDNA impairs trophoblast function via NF-κB signaling pathway. (A) Flow cytometry analysis of apoptosis levels in HTR-8/SVneo cells pretreated with or without JSH-23 for 6 h, then treated with mtDNA (5ng/ml) for 24 h. (B-C) Scratch wound healing assay (B) and transwell assay (C) evaluating the invasive and migratory capacities of HTR-8/SVneo cells under the indicated conditions. Data are presented as mean ± SD, n=3. Statistical significance was determined by one-way ANOVA; ns: not significant, *$p < 0.05$, **$p < 0.01$, ***$p < 0.001$.

Supplementary tables

Table S1. Clinical characteristics of the participants.

**Category**	**Normal (n = 30)**	**URPL (n = 30)**	***p*-value**
Age (years)	33.1 ± 3.6347	31.4 ± 4.8351	0.3868
Days of gestation	50 ± 2.1082	56.5 ± 11.3944	0.1077
Number of miscarriages	0.7±0.6749	2.1±0.3162	<0.0001

Values are shown as the mean ± SD; Student's t-test.

Table S2. Primer sequences.

**Name**	**Sequence (5'-3')**
CISH-Forward	*CCAGCCCAGACAGAGAGTGAG*
CISH-Reverse	*ATACCAGCCAGATTCCCGAAGG*
CYP1A1-Forward	*CAAGGGGCGTTGTGTCTTTG*
CYP1A1-Reverse	*GTCGATAGCACCATCAGGGG*
CGAS-Forward	*GAAGGCCTGCGCATTCAAAA*
CGAS-Reverse	*GTGAGAGAAGGATAGCCGCC*
IL6-Forward	*GCCTTCGGTCCAGTTGCCTTC*
IL6-Reverse	*GTTCTGAAGAGGTGAGTGGCTGTC*
IL33-Forward	*CTCCGCTCTGGCCTTATGAT*
IL33-Reverse	*AAGGCAAAGCACTCCACAGT*
CXCL10-Forward	*AGCAGAGGAACCTCCAGTCT*
CXCL10-Reverse	*ATGCAGGTACAGCGTACAGT*
ATP6V1A-Forward	*TTATGAACGAGCAGGCAGGG*
ATP6V1A-Reverse	*CACCAGGTGGAGAAACTGCT*

Table S3. The antibodies used for western blotting, immunofluorescence staining, co-immunoprecipitation, and flow cytometry.

**Antibody identifier**	**Product identifier**	**Source**	**RRID**
Anti-CD68	GB113150	Servicebio	AB_2924885
Anti-AHR	83200S	CST	AB_2800011
FITC anti-human CD14	982502	BioLegend	AB_2616906
APC-Cy7 anti-human CD45	304014	BioLegend	AB_314402
PE-Cy7 anti-human AHR	25-5925-82	Thermo Fisher Scientific	AB_2573501
Anti-F4/80	GB113373	Servicebio	AB_2938980
Anti-cGAS	79978	CST	AB_2905508
Anti-p-STING	50907	CST	AB_2827656
Anti-STING	13647	CST	AB_2732796
Anti-p-TBK1	5483	CST	AB_10693472
Anti-TBK1	38066	CST	AB_2827657
Anti-p-IRF3	37829	CST	AB_2799121
Anti-IRF3	4302	CST	AB_1904036
Anti-β-ACTIN	66009-1-Ig	Proteintech	AB_2687938
Anti-dsDNA	ab27156	Abcam	AB_470907
Anti-TOMM20	F3252	Selleck	AB_3698476
Anti-LAMP1	9091	CST	AB_2687579
Anti-ATP6V1A	ab199326	Abcam	AB_2802119
Anti-CISH	8731	CST	AB_11178524

Anti-Ubiquitin	43124	CST	AB_2799235
Anti-LC3B-I/LC3B-II	2775	CST	AB_915950
Anti-TLR9	A5027	Selleck	AB_3698477
Anti-NLRP3	F0335	Selleck	AB_3698478
Anti-p-NF-κB (P65)	3033	CST	AB_331284
Anti-NFκB P65	8242	CST	AB_10859369
Anti-p-IKBα	2859	CST	AB_561111
Anti-IKBα	A5599	Selleck	AB_3698479
Anti-BCL2	15071	CST	AB_2744528
Anti-Activated Caspase 3	ET1602-47	HUABIO	AB_3069660
Anti-MMP9	F0008	Selleck	AB_3696822
Anti-MMP2	40994	CST	AB_2799191
APC anti-human CD206 (MMR) Antibody	321110	Biolegend	AB_571885
PE anti-human CD86 Antibody	374206	Biolegend	AB_2721633
PE-conjugated anti-HLA-G antibody	ab24384	Abcam	AB_448029
